# Biodiversity Impact Assessment Considering Land Use Intensities and Fragmentation

**DOI:** 10.1021/acs.est.3c04191

**Published:** 2023-11-16

**Authors:** Laura Scherer, Francesca Rosa, Zhongxiao Sun, Ottar Michelsen, Valeria De Laurentiis, Alexandra Marques, Stephan Pfister, Francesca Verones, Koen J. J. Kuipers

**Affiliations:** † Institute of Environmental Sciences (CML), Leiden University, 2333 CC Leiden, The Netherlands; ‡ Institute of Environmental Engineering, ETH Zurich, 8093 Zurich, Switzerland; § College of Land Science and Technology, China Agricultural University, Beijing 100083, China; ∥ Department of Industrial Economics and Technology Management, Norwegian University of Science and Technology (NTNU), 7491 Trondheim, Norway; ⊥ European Commission-Joint Research Centre, 21027 Ispra, Italy; # PBL Netherlands Environmental Assessment Agency, 2500 GH The Hague, The Netherlands; ∇ Industrial Ecology Programme, Department for Energy and Process Engineering, Norwegian University of Science and Technology (NTNU), 7491 Trondheim, Norway; ○ Department of Environmental Science, Radboud Institute for Biological and Environmental Sciences (RIBES), Radboud University, 6525AJ Nijmegen, The Netherlands

**Keywords:** biodiversity loss, characterization factors, ecosystem quality, extinction risk, land occupation, land transformation, life cycle assessment, species richness

## Abstract

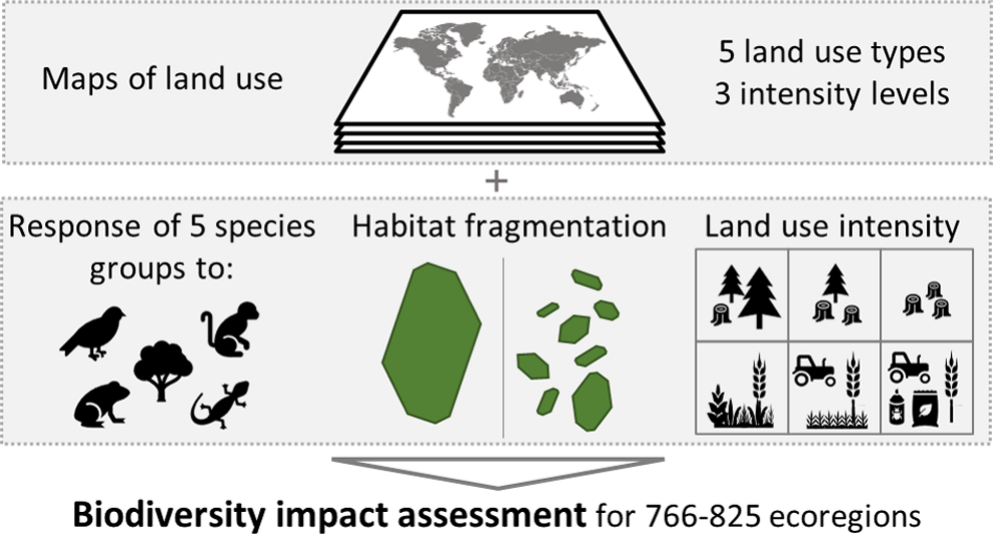

Land use is a major threat to terrestrial biodiversity. Life cycle assessment is a tool that can assess such threats and thereby support environmental decision-making. Within the Global Guidance for Life Cycle Impact Assessment (GLAM) project, the Life Cycle Initiative hosted by UN Environment aims to create a life cycle impact assessment method across multiple impact categories, including land use impacts on ecosystem quality represented by regional and global species richness. A working group of the GLAM project focused on such land use impacts and developed new characterization factors to combine the strengths of two separate recent advancements in the field: the consideration of land use intensities and land fragmentation. The data sets to parametrize the underlying model are also updated from previous models. The new characterization factors cover five species groups (plants, amphibians, birds, mammals, and reptiles) and five broad land use types (cropland, pasture, plantations, managed forests, and urban land) at three intensity levels (minimal, light, and intense). They are available at the level of terrestrial ecoregions and countries. This paper documents the development of the characterization factors, provides practical guidance for their use, and critically assesses the strengths and remaining shortcomings.

## Abstract 2

Land use is a key driver of terrestrial biodiversity loss. We developed a new impact assessment indicator for assessing its impacts on species richness across the world’s terrestrial ecoregions.

## Introduction

For terrestrial species, land use and land use change are the most important pressures that lead to species loss,^1−3^ with on average a 13.6% reduction in species richness due to land use^4^ and wilderness areas being continuously lost.^5^ The main reason is that habitat area gets lost, fragmented, and degraded.^6^

It is, therefore, crucial to be able to assess the impacts resulting from land use and land use change to work toward minimizing and halting these impacts and to avoid trade-offs between impacts or between regions. One widely used tool to support environmental decision-making and assess such impacts is life cycle assessment (LCA).

LCA is a very dynamic and comparably young research field with many new developments and few established standards. The Life Cycle Initiative hosted by UN Environment, therefore, launched the GLAM project (Global Guidance for Life Cycle Impact Assessment Indicators and Methods) in 2013 with the aim of providing consensus on which life cycle impact assessment indicators to use. After two previous rounds of recommendations focused on different impact categories, areas of protection, and cross-cutting issues,^7,8^ GLAM is now in its third phase. GLAM3 aims to consolidate and update recommendations for assessing impacts on several impact categories related to human health, natural resources, and ecosystem services as well as ecosystem quality. For ecosystem quality, the focus is on indicators at the endpoint level, expressed in potentially disappeared fractions of species (PDF) as a measure of the relative loss in species richness.^9,10^ This work will be considered in the upcoming recommendations regarding models for ecosystem quality, covering the indicator for assessing land use impacts on an endpoint level.

This impact category has been continuously developed over the last decades, with the first operational indicators available in the early 2000s.^11^ These early indicators did not distinguish impacts spatially, nor did they include individual species groups, sometimes even using biodiversity proxies such as net primary productivity or the degree of naturalness, without connection to species richness, which is, due to pragmatic reasons, the currently recommended metric to use in LCA.^10^ Indirectly, they already applied the concept underlying a species-area relationship (SAR), in which biodiversity gets lost with increasing land use.^12,13^ Since then, approaches for land-use-related impacts have been expanded and greatly improved. Spatial and taxonomic detail have been introduced,^14^ and more complex versions of the SAR have been developed. The matrix-calibrated SAR,^15^ for example, considers the different sensitivities of species to different parts of the matrix (i.e., the human-modified habitat) as opposed to the classic SAR, which assumes that no species can survive in human-modified land. The countryside SAR^16^ similarly accounts for species’ affinity to habitat and the fact that species may be able to survive in the absence of natural habitat and was shown to outperform the matrix-calibrated SAR at least in some cases.^17^ In GLAM, land use was recognized as a priority area early on,^18^ and a task force was mandated in 2014 to identify the best available indicators to assess biodiversity loss related to land use activities within GLAM1.^19^ At the time, an interim recommendation for land use was based on Chaudhary et al.^16^ and included in the report of the first set of recommendations.^7^

In the years since, indicator development has continued to also include land use intensities^20^ or the fragmentation of the landscape,^21,22^ which are both considered important.^19^ While the consideration of land use intensities is straightforward if the necessary data are available, the consideration of fragmentation requires a refinement of the model. Two different concepts can play a role here: the equivalent connected area (ECA) and the metapopulation capacity. The ECA is the area of a single habitat patch providing a probability of connectivity equivalent to the actual habitat pattern that may be fragmented.^23^ In contrast, the metapopulation capacity is the ability of a fragmented landscape to support a group of populations of the same species,^24^ and this measure has been integrated into a so-called species-fragmented area relationship.^25^ Both concepts have previously been combined with the countryside SAR to develop characterization factors (CFs).^21,22^ However, the latter concept relies on additional parameters related to migration and extinction rates that are difficult to estimate,^25^ and the corresponding CFs are, therefore, more limited in their spatial and species-group coverage.^22^ None of the existing indicators simultaneously consider land use intensities and fragmentation, which is the goal of the further indicator development presented here.

## Methods

### Characterization Factor Framework

Land use CFs quantify the per-area effects of different land use classes on species richness, i.e., the number of species. Figure S1 depicts a conceptual overview of the methodology followed to derive the CFs. After starting off with the overall regional impacts, there are two approaches for converting these to impacts per unit of pressure (here, per area of a certain land use class): average and marginal.^26^ Both approaches build on the species–habitat relationship (eq 1) to estimate relative species loss (RSL, dimensionless, but for clarity and by convention, the unit is called PDF) specific to species group *g* (amphibians, birds, mammals excluding bats, reptiles, or plants) and ecoregion *j* (766 in total). They do so either directly (eq 2) or through its partial derivative (eq 3) and consider the total regional land use area (*A*_lu_, m^2^) and an allocation factor (*a*, dimensionless) to attribute impacts to specific land use types *i* (cropland, pasture, plantations, managed forests, or urban land)^21,27^ and intensities *m* (minimal, light, or intense). Compared to land occupation (occ) CFs (expressed in PDF/m^2^), land transformation (tra) CFs (expressed in PDF·yr/m^2^) additionally account for the regeneration time^28^ (*t*, yr) of species group *g* in region *j* and land type *i* (eq 4).^29^ Combining the regional characterization factors with the corresponding global extinction probabilities (GEPs, dimensionless) transforms the regional PDF (i.e., relative species extirpations) to global PDFs (i.e., relative global species extinctions) (eq 5).^30^ The PDFs refer to potential relative species losses in the long term if the land-use activities are sustained; thus, they go beyond imminent losses and account for extirpation/extinction debts.^31^
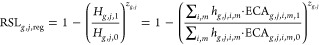
1
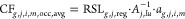
2
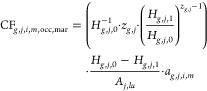
3
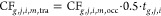
4
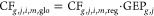
5

The species–habitat relationship (eq 1) enhances the countryside SAR. It accounts for the habitat affinity (*h*, dimensionless) of species to a certain natural or human-modified habitat type *i* and intensity level *m* and the change in the equivalent connected area (ECA, m^2^) of the habitat between a reference state (subscript 0) and the current global land use configuration (subscript 1). Their products’ sum yields the suitable connected area (*H*, m^2^), retaining subscripts 0 and 1. Because we assume a fully natural reference state (with an affinity of 1), *H*_*j,0*_ equals the area of the ecoregion (*A*_*j*_, m^2^). *z* (dimensionless) denotes the slope of the relationship.

The ECA allows one to consider habitat fragmentation. It equals the total area (*A*, m^2^) of the land type *i* and intensity *m* if all patches are connected and approaches the area of the largest single patch if all patches are fully disconnected. The connectivity of the patches depends on the probability of dispersal for the species group *g* in region *j* and land type *i* and intensity *m* between the pair of patches *x* and *y* (eq 6).^23^
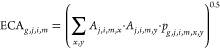
6

The probability of dispersal (*p*, dimensionless), in turn, is based on, among others, the median dispersal distance of species group *g* in region *j* and the resistance (*r*, dimensionless) of the surrounding landscape separating the patches. See further details on the probability of dispersal in the Supporting Information and on the resistance and habitat affinity under the model parametrization for plants and vertebrates.

### Model Parametrization for Plants

We used the local monitoring and experimental data collected by Gallego-Zamorano et al.^32^ and followed their meta-analytical approach to estimate the relative local plant species richness (*rr*, dimensionless) due to land use (Table S1) as a basis for habitat affinities (eq 7). While generally distinguishing three land use intensity levels, we distinguished only two intensity levels for plantations because the absolute difference between plantations with a minimal or light intensity was below 0.05, which is practically insignificant and statistically insignificant according to Welch’s *t* test applied to the model estimates and standard errors of the model before merging any intensity classes.
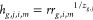
7

Biome-specific *z*-values for plants were taken from Table 2 in Gerstner et al.^33^

The dispersal distance per ecoregion represents the median maximum dispersal distance of the plant species present in an ecoregion. The presence of 26,573 vascular plant species was derived from plant species distributions based on the best-performing Maxent prediction from Borgelt et al.^34^ and the highest threshold at which no occurrence record would be omitted. The plant species’ maximum dispersal distance was estimated based on the linear regression model by Tamme et al.,^35^ considering the categorical traits “dispersal syndrome” and “growth form” as fixed effects. The traits were obtained from the TRY plant trait database.^36^ Both traits were available for 3245 plant species, for which the dispersal distance could then be estimated using the linear regression model. Any gaps were filled based on the taxonomy, taking the average maximum dispersal distance of either the genus, family, order, class, or, as a last resort, the kingdom.

The landscape resistance (*r*) ideally considers the species overlap between a habitat patch and its surrounding landscape of different land use classes *k*. This information is not available for plant species. Therefore, it was estimated based on the relative species richness (*rr*) as follows
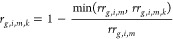
8where *g* is the species group of plants, *i* is the land use type of the habitat, *m* is the intensity level, and *k* refers to the surrounding landscape.

The global extinction probabilities of vascular plants were obtained from Verones et al.,^30^ who also used the plant species distributions from Borgelt et al.^34^ as a basis for their analysis.

See further details in the Supporting Information.

### Model Parametrization for Vertebrates

The inclusion of the effect of land use intensities on vertebrates in the model was based on two sources: (i) the PREDICTS database^37^ for all land use types except managed forests and (ii) a meta-analysis on managed forests.^38^

Using the PREDICTS database,^37^ our aim was to replicate the results for relative species richness of Newbold et al.,^4^ filtering out all species not belonging to the *Animalia* kingdom. We used the animal data as a proxy of vertebrates, as they ensured a larger sample size and proved to be more robust when the analysis was performed. Using the function GLMER (generalized linear mixed-effects model) from Newbold’s^39^ library StatisticalModels, we applied the same model as in the section “Statistical model structure” in the Supporting Information of Newbold et al.^4^ for species richness of different land use types and intensities.

The meta-analysis by Chaudhary et al.^38^ was selected as a data source for managed forests, as it provided more comprehensive coverage of related species richness responses than PREDICTS.^37^ To be consistent with what was done for the other land use types, the species groups selected were those belonging to the *Animalia* kingdom. For each data point, the relative species richness was obtained as the ratio between values in managed forest sites and reference (unmanaged) forest sites. To differentiate between the three intensity levels, specific forest practices were selected according to the availability of data and the approach by Chaudhary and Brooks.^20^ The median of the relative species richness for each forest management intensity level was calculated.

As for plants, some intensity levels were merged for some land use types due to insignificant differences: plantations light and intense, cropland light and intense, and pasture light and intense.

Where habitat affinities could be estimated at the ecoregion level for broad land use types based on the number of species (*S*) from IUCN^40^ occurring in them (only applies to vertebrates for all land use types, except for managed forests), they were rescaled to account for land use intensities based on globally defined scaling factors (*f*, dimensionless, eq 8), similar to Chaudhary and Brooks.^20^ Such scaling factors were derived for each land use type *i* from the ratio of relative species richness for intensity level *m* relative to a minimal land use intensity. For managed forests, the same approach as that for plants was used (eq 7). Biome-specific *z*-values for vertebrates were taken from Storch et al.^41^
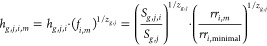
9

Following Kuipers et al.,^21^ dispersal distances were derived from the body mass extracted from the EltonTraits database^42^ for birds and mammals, and a dispersal distance of 9 km was assumed for amphibians and reptiles.

Concerning landscape resistance, the following formula was mostly used, which is adapted from Kuipers et al.^21^ to integrate land use intensities again
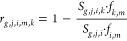
10

As for the habitat affinities, a modified approach was needed when the habitat type or the surrounding landscape was a managed forest (eq 8). If one habitat type of such a pair is not a managed forest, its relative species richness can be back-calculated by solving eq 7 for *rr*, which is then ecoregion-specific.

See further details in the Supporting Information.

### Model Parametrization Regarding Land Use and Intensity

We aligned the definition of land use intensity with that proposed by Newbold et al.^4^ (Table S2). We used land use maps from the HIstoric Land Dynamics Assessment + (HILDA+) in the year 2015^43^ as the base map. HILDA+ harmonizes multiple remote sensing data with national land-use inventories. The data set is one of the latest global land-use maps, has a high resolution (about 30 × 30 arcseconds), and covers a long period (from 1960 to 2019). It includes six land use types: urban, cropland, pasture/rangeland, forest, unmanaged grass/shrubland, and sparse/no vegetation. To reduce the computational requirements of determining the equivalent connected area, we aggregated the land use data to 5 arcminutes based on the most frequent category within the larger grid cell.

The reference land use was primary vegetation, for which we considered unmanaged grass/shrubland and sparse/no vegetation from HILDA+, as well as forest from HILDA+ if it overlapped with “Naturally regenerating forests without any signs of management, including primary forests” as the most frequent category among the forest management classes from Lesiv et al.^44^

Various data sets were used to define intensity levels for the five anthropogenic land uses of interest (see also the Supporting Information). For cropland, we considered the area equipped for irrigation^45^ and phosphorus and nitrogen fertilizer use.^46^ For pasture, we used categorical data from GLOBIO4.^47^ For plantations, we used categorical forest management^44^ and oil palm plantation data.^48^ For managed forests, we considered forest extent loss.^49^ Finally, for urban areas, we used the categorical Global Human Settlement data.^50^

### Taxonomic and Spatial Aggregation

CFs for species groups *g* were aggregated in two steps: first at the kingdom level and then altogether (eq 11). All animal (in our case vertebrate) species groups *v* were averaged, giving equal weight to each species group, irrespective of its number of species (option 2 in ref (51)). Afterward, plant and average animal CFs were averaged, again giving equal weight to each species group.
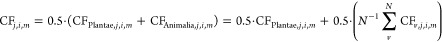
11

CFs representing relative regional or global species losses at the ecoregion *j* level were aggregated to country *c* and global levels by the average of the ecoregion CFs (in the country) weighted by the area of the ecoregions (within the country) (eq 12).^21^ As such, CFs at the aggregated spatial scales still represent relative regional or global losses at the ecoregion level; just that the ecoregion affected remains unclear. For global aggregation, we provide an alternative set of CFs weighted by the area of the land use type and intensity. This weighting is more accurate, as demonstrated in a test application to global land occupation in 2015, but differences are rather small (Table S3), and when considering proxies for areas where a certain land use currently does not exist, only the ecoregion area can be used.
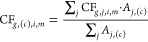
12

To assess spatial uncertainties due to aggregation from ecoregions to countries or the globe, the relative weighted standard deviations were calculated.

### Comparison with Existing Characterization Factors and IUCN Data

Since our CFs build on concepts from Chaudhary and Brooks^20^ and Kuipers et al.^21^ and are intended to succeed the CFs that received an interim recommendation in GLAM1,^16^ we compared our CFs to theirs. We compared only CFs calculated based on an average approach, as Chaudhary and Brooks^20^ do not provide marginal CFs. Moreover, we compared regional species loss instead of global species loss to focus on differences because of the underlying species–area or species–habitat relationship and model parametrization rather than differences in the translation of regional species loss to global species loss. Global species loss is less comparable because the global extinction probabilities used here and by Kuipers et al.^21^ sum to 1 globally for each species group, while the vulnerability scores used by Chaudhary and Brooks^20^ sum to a different arbitrary value for each species group. To convert the CFs from Chaudhary and Brooks^20^ for global absolute species loss to regional relative species loss, we divided them by the total species richness and vulnerability score of an ecoregion and species group. Where land use classes are broader and consider no or fewer intensity levels, CFs were duplicated to match the number of classes of the CFs with a finer classification. The CFs from GLAM1 could only be compared for aggregated species groups, as the unit is consistent only then. Moreover, only CFs for global species loss are available in GLAM1, without data allowing for a conversion back to regional species loss.

We compared the CFs based on two statistical measures: the Spearman correlation coefficient and the percent bias. The Spearman correlation coefficient is nonparametric, considering the ranks of the data instead of the actual values. It ranges from −1 to 1, with values closer to the boundaries indicating a stronger negative or positive monotonic relationship. The percent bias reflects the average tendency of one set of values to be smaller or larger than another set of values. We used the newer CFs as the reference so that a negative percent bias implies that the older CFs are smaller, while a positive percent bias implies that the older CFs are larger.

Apart from comparing our CFs to other CFs, we also compared our estimates of the total number of species lost globally with the number of species threatened by land use according to the IUCN Red List.^40^ Species were considered threatened if the threat category would be vulnerable, endangered, critically endangered, extinct in the wild, or extinct, excluding data deficient, least concern, and near threatened. They were considered threatened specifically by land use if some of the threats species faced were classified by IUCN as 1 (residential and commercial development), 2.1 (annual and perennial nontimber crops), 2.2 (wood and pulp plantations), or 2.3 (livestock farming and ranching) and if the criteria used to determine the threat category were not A1–A4 based on only d or e, which are related to exploitation, introduced taxa, pollutants, etc.

### Sensitivity Analysis

We conducted a sensitivity analysis to test how certain data and modeling choices affect the resulting CFs. For this purpose, we tested three cases: (1) considering lower and upper estimates for the relative change in species richness due to land use and its intensity, (2) disregarding land use intensity levels, and (3) disregarding land fragmentation.

The lower and upper bounds of the relative species richness represent the 95% confidence intervals, an additional output from the respective statistical models for plants and vertebrates based on the PREDICTS data. Since a different data set and approach were used for the impacts of managed forests on vertebrates, the 2.5th and 97.5th percentiles were used as the upper and lower bounds.

To only consider broad land use types without distinguishing land use intensities, the relative species richness for plants and, in the case of managed forests, for vertebrates was recalculated as described above after pooling the data for different land use intensities of a certain broad land use type. In other cases regarding vertebrates, the ecoregion-level habitat affinities of broad land use types were simply not rescaled to account for land use intensities.

To remove the fragmentation effects from the CF model, the equivalent connected area was replaced with the total habitat area available in an ecoregion.

We used the same statistical metrics as above for the comparison between the default CF model and the sensitivity cases: the Spearman correlation coefficient and the percent bias. However, here, we compared the regional species loss at the ecoregion level instead of the CFs for different land uses within an ecoregion. It is possible here because the overall land use area is the same in all cases.

A contribution-to-variance analysis was conducted to identify which factors seem to influence the resulting CFs the most. It is estimated based on the squared Spearman correlation coefficient of one factor relative to the sum of squared Spearman correlation coefficients of all factors considered.^52^ As factors, we considered (1) the habitat affinity (h), which is influenced by both the species group and land use class, (2) the ecoregion area, (3) the area used for human activities relative to the ecoregion area (the more land is used, the more impact additional land use has), (4) the equivalent connected area (ECA) relative to the area used (the higher, the more fragmented the area), (5) the *z*-value, and (6) the global extinction probability (GEP).

Finally, we calculated the correlation between CFs of different species groups to assess their complementarity.

### Proxies to Reach Full Ecoregion and Country Coverage

If the same land use type is available at a different intensity level, then the relative local species richness for different intensity levels from Table S1 was used for rescaling the CFs. If the entire land use type is missing, the regional CFs from ecoregions within the same biome were averaged and multiplied by the GEP of the ecoregion of interest (eq 5). If the GEP is missing, the GEPs from ecoregions within the same biome were divided by the ecoregions’ areas, averaged, and then multiplied with the area of the ecoregion of interest. By doing so, all 825 terrestrial ecoregions could be covered. Only plantations are missing in biomes 9 (flooded grasslands and savannas) and 11 (tundra).

Two island countries or territories could not be matched with ecoregions. In this case, we identified the three nearest neighboring countries or territories and took a simple average. The standard deviations representing the spatial uncertainties were then pooled.

## Results

### Spatial Distribution of Characterization Factors

**Figure 1:**
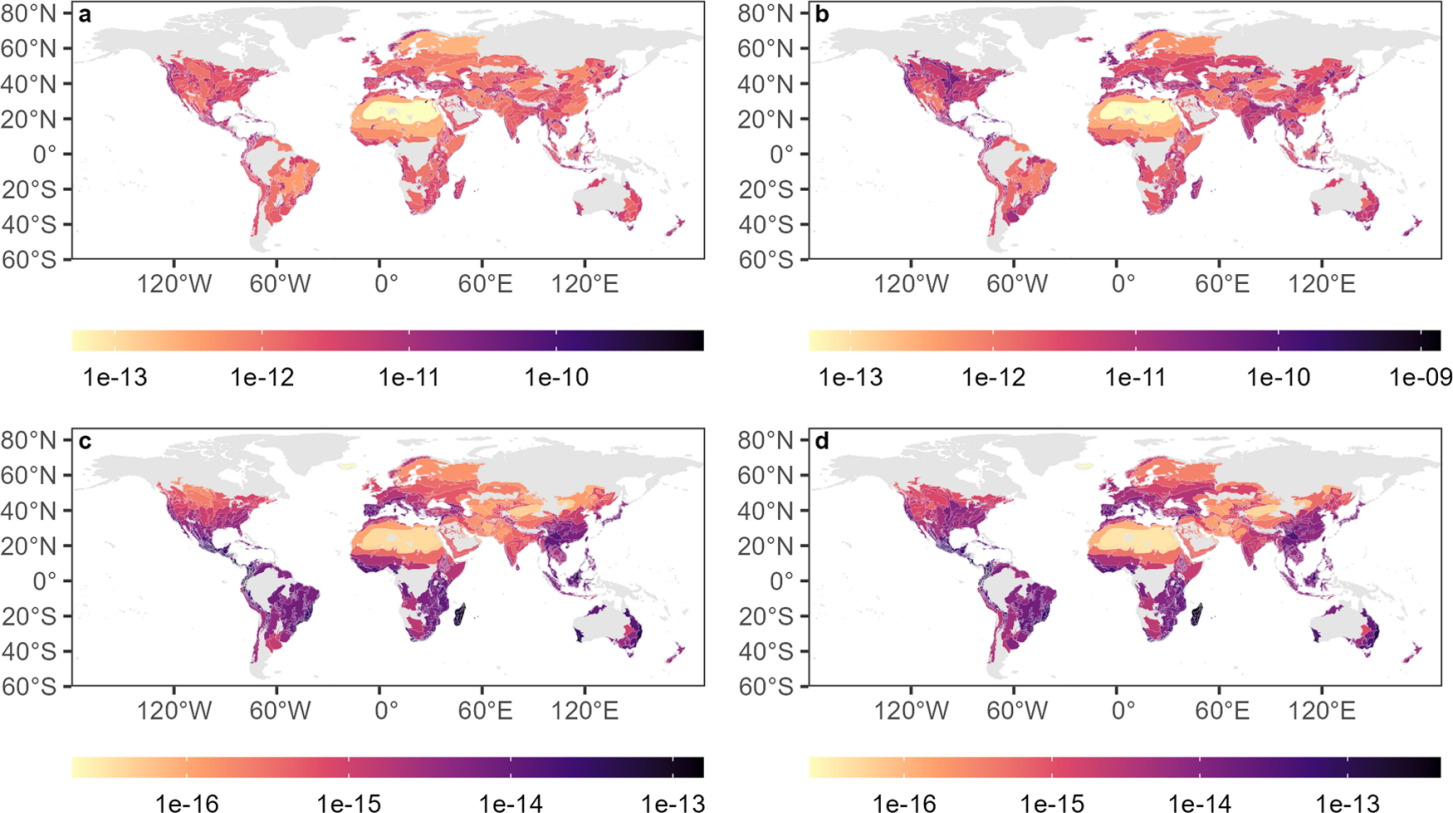
Land occupation characterization factors at the ecoregion level for cropland with intense use and potential impacts on plant species richness as an example. The unit is PDF/m^2^. (a) Average impacts on regional species richness, (b) marginal impacts on regional species richness, (c) average impacts on global species richness, and (d) marginal impacts on global species richness. Gray denotes no data, indicating either the absence of the specific land use class in these regions or missing species data. More characterization factors are available through the use of proxies.

The ecoregion-level CFs show great spatial variation with values ranging over several orders of magnitude. As an example, land occupation CFs for plant species for the land use class cropland with intense use vary over about 4 orders of magnitude regardless of the calculation approach adopted and both in the case of regional and global species richness (Figure 1). The highest impacts on global species richness (average approach) occur in Madagascar lowland forests, where plants have the highest global extinction probability. The highest impacts on regional species richness (average approach) instead occur in the Caribbean’s Enriquillo wetlands, which is the smallest ecoregion with intensely used cropland and has a relatively high *z*-value. In contrast, the lowest relative global species loss occurs in Iceland boreal birch forests and alpine tundra, where plants have the lowest global extinction probability, the lowest *z*-value, and a relatively small share of anthropogenic land use. The lowest relative regional species loss occurs in the Sahara desert, the largest terrestrial ecoregion with a tiny share of anthropogenic land use. The Supporting Information contains similar maps for the remaining land use classes and species groups (Figures S2–S26).

### Characterization Factors for Different Approaches, Species Groups, and Land Use Classes

**Figure 2:**
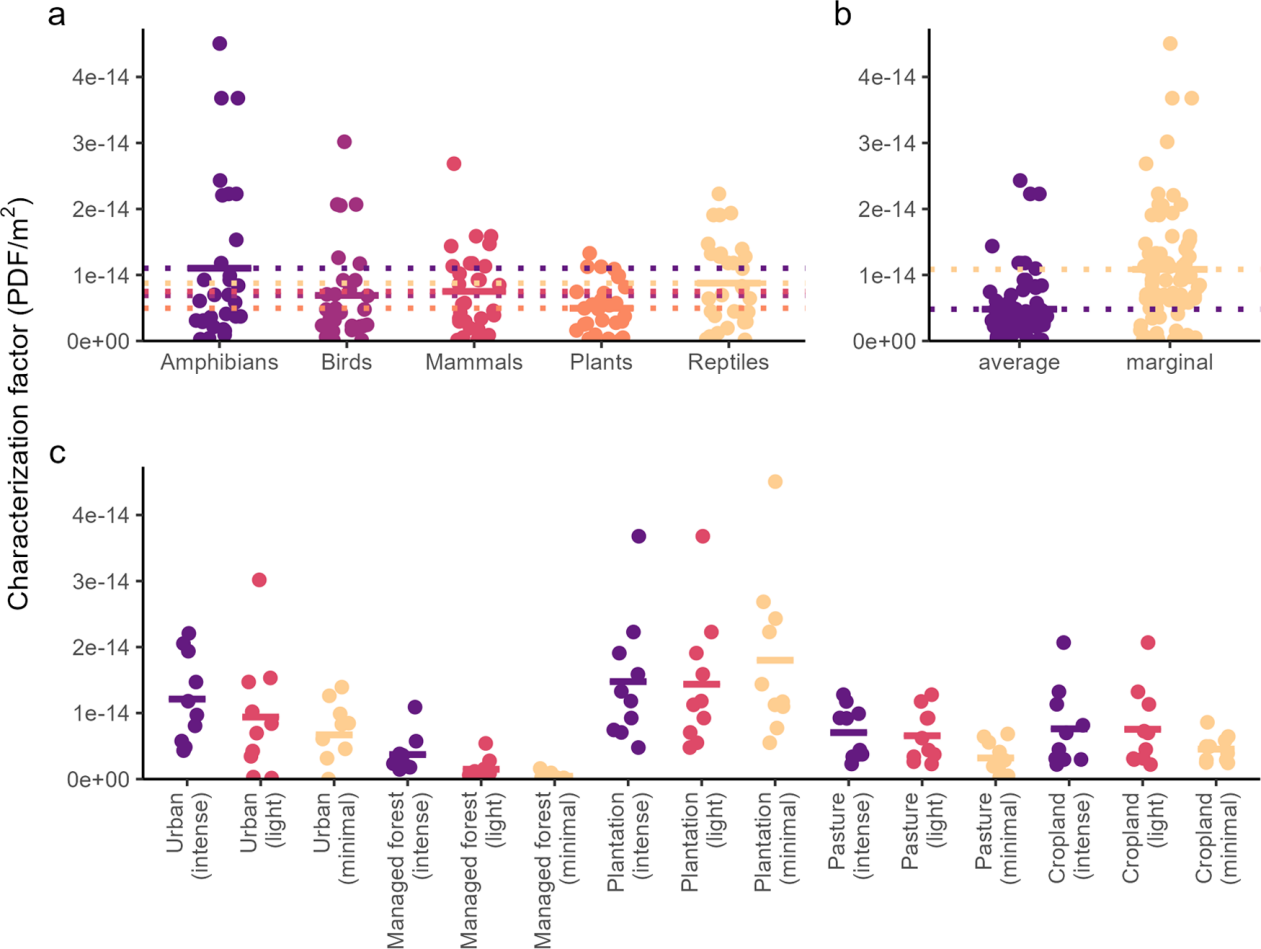
Globally aggregated land occupation characterization factors for global species loss for different (a) species groups, (b) approaches, and (c) land use classes. The ecoregion-level CFs were weighted by land use area. Colors are for visualization purposes but are not needed for interpretation.

When looking at globally aggregated CFs (Figure 2), there are noticeable differences among the different sets of CFs. CFs for global species loss using a marginal approach are more than twice as high as when using an average approach. This highlights the accelerating species loss with increasing land use, as expected from the power law underlying the species–habitat relationship.

The CFs for amphibians are, on average, the highest, followed by those for reptiles, possibly because these two have the highest average *z*-values. In contrast, they tend to be lowest for plants, possibly because they have the highest average habitat affinities. However, such rankings among species groups also depend on land use type and intensity. Birds show the largest difference between average and marginal CFs, with a factor of almost 10.

More intense land use leads to larger potential species losses than light use, which, in turn, leads to higher losses than minimal use. However, differences are sometimes small. For globally aggregated CFs, only minimally used plantations deviate from this expected trend and exhibit higher CFs than lightly or intensely used plantations. At the ecoregion level, the CFs for plantations still follow expectations across the intensity levels. This suggests that the deviation stems from the spatial distribution of the intensity levels for plantations, meaning that plantations with a minimal use intensity are more likely to occur in ecoregions with an overall higher relative species loss (Figures S8 and S20). Regarding land use types, plantations generally have the highest CFs, and managed forests, the lowest. Applying the CFs to global land occupation in 2015 suggests that almost 16% of terrestrial plant and vertebrate species face a risk of extinction in the long term (Table S3).

### Comparison with IUCN Data and Other Indicators

We found strong positive correlations between our newly developed CFs and the ones presented in GLAM1,^16^ Chaudhary and Brooks,^20^ and Kuipers et al.^21^ (Table S4). The latter also show a similarly strong correlation with each other. The percent bias between the CFs from Kuipers et al.^21^ and our CFs is negative across all species groups, indicating that their CFs are lower than ours. These results are not surprising since our CFs consider more anthropogenic land use, also including managed forests, and their intensity levels. In the case of Chaudhary and Brooks,^20^ the results are more mixed across species groups, and the percent biases are sometimes negative and sometimes positive.

Although our CFs are always higher than those of Kuipers et al.,^21^ there is no indication that ours represent an overestimation of the biodiversity impacts. Our estimates of the total number of species lost globally (*n*_threat_) are always considerably lower than the number of species threatened by land use according to the IUCN Red List (*n*_threat,IUCN_) (Table S4). Lower results are in line with expectations since IUCN’s data inform about threats that do not necessarily end in the extinction of the species as considered in our CFs. Moreover, although land use poses a threat to all species from IUCN considered here, other threats can also play a role, whereas our CFs focus solely on land use. The difference is highest for amphibians (factor of almost 9), but in this case, our CFs have a very similar magnitude to those from Chaudhary and Brooks,^20^ with a percent bias of only 7%, and the differences would be even larger for Kuipers et al.,^21^ where amphibians show the largest absolute percent bias with −46%. The difference is the lowest for birds (factor of less than 2). In this case, our CFs are relatively close to the CFs of Kuipers et al.,^21^ with a percent bias of only −12%. In contrast, the CFs of Chaudhary and Brooks^20^ are much higher for birds, with a percent bias of 104%, which seems unrealistic, as the number of species potentially going extinct would then be very similar to and even higher than the number of species threatened.

### Sensitivity Analysis

All sensitivity analyses performed on different variants of the CFs show very strong positive correlations (Table S5). The regional species loss at the ecoregion level obtained with the lower bound of the relative species richness due to land use is higher than the default regional species loss for all species groups, and the opposite applies to the upper bound of the relative species richness. If no distinctions between land use intensities are considered, then the difference in the magnitude is very small, with a percent bias ranging from −2 to 6%. When fragmentation is not considered, regional species losses across the different species groups are lower than those in the default model. This is expected, given that the remaining suitable habitat area can only remain the same or reduce when considering the connectivity among different habitat patches. Overall, the model appears to be more sensitive to the choice to consider fragmentation than to consider land use intensities.

The contribution-to-variance analysis (Table S6) showed that the CFs for global species loss are most sensitive to global extinction probabilities and habitat affinities. Global extinction probabilities play the key role for amphibians and reptiles, and the habitat affinities for birds and mammals, and both are similarly important for plants. Where global extinction probabilities play a bigger role, the location of land use matters more than the land use class and vice versa. The slopes of the species–habitat relationship, the ecoregion areas, the shares of anthropogenic land use, and the degree of fragmentation of that land use are less decisive.

The correlation of CFs among the different vertebrate groups is high, ranging from 0.78 to 0.87. In contrast, it is low between plants and any of the vertebrate groups, ranging from 0.21 to 0.38 (Table S7). This observation highlights the complementarity of considering plants and the importance of considering species groups that differ at taxonomic ranks higher than just classes, as in the case of different vertebrate groups.

## Discussion

### Limitations and Advancements

In GLAM1, Curran et al.^11^ made seven best-practice recommendations for the development of characterization factors for impacts of land use on biodiversity, ordered by priority. First, a multidimensional approach should be used, going beyond species richness and covering multiple taxonomic groups. We only consider impacts on species richness in this paper, as is still common practice in LCA. On the one hand, species richness requires less data to be collected than some other biodiversity metrics by only relying on presence data, and it is a simple metric that is intuitive to understand. Additionally, species richness exhibits some desirable properties for biodiversity monitoring, like being sensitive to community changes and yielding consistent responses across replicates.^53^ On the other hand, it cannot send early warning signals of biodiversity loss,^53^ but this is not relevant to our indicators that reflect potential long-term losses. Moreover, the information it conveys about biodiversity is limited, and it is advised to use it within a set of a few complementary metrics to provide a more comprehensive picture of biodiversity.^53^ However, abundance-related metrics have only been used on a small scale in life cycle impact assessment (LCIA) so far.^54^ Similarly, indicator development for functional diversity is still in its infancy, and no operational, global model exists yet.^55^ While relying on a single biodiversity metric, we cover five species groups: amphibians, birds, mammals, reptiles, and plants. As the correlation analysis has shown, the inclusion of plants is especially important given their complementarity to the four vertebrate groups. The inclusion of plants goes beyond the two recent models on which we build that either do not consider plants^21^ or use taxon-generic z-values and relative species richness estimates,^20^ whereas ours are plant-specific. The representativeness of the species within the included species groups remains unclear. For example, the median dispersal distances and global extinction probabilities cover almost 27,000 vascular plant species, whereas there are about 308,000 known vascular plant species as of 2016.^56^ Ideally, future models will also cover invertebrates, such as insects, which have only been covered in case studies so far.^57^

Second, models should develop not only local but also regional components and document both. We do so by applying the species–habitat relationship that translates local to regional species losses. The relative local species richness of the plants can be found in Table S1.

Third, the CFs should reflect biodiversity’s intrinsic value and vulnerability. This information is captured within the global extinction probabilities that consider endemism and threat levels.^30^

Fourth, CFs should differentiate basic land use intensities. We distinguish three intensity levels: minimal, light, and intense. This constitutes an addition compared to Kuipers et al.^21^ Instead of considering only taxon-generic effects of land use intensities like Chaudhary and Brooks,^20^ we distinguish the effects on plants and animals (not specifically vertebrates). However, as in Chaudhary and Brooks,^20^ the effects of land use intensities are only considered at a global level and not the ecoregion level, and the relative species richness used as a basis entails high uncertainties. Moreover, differences between intensity levels are sometimes small (Figure 2).

Fifth, uncertainties should be assessed and reported. While we do not provide uncertainty ranges for the CFs at the native scale, which would be challenging due to the computational requirements of the model especially for the consideration of fragmentation (an advancement compared to Chaudhary and Brooks^20^), we conducted several sensitivity analyses, compared our CFs to previous CFs and IUCN data, and are transparent about the limitations of our CFs. Additionally, we assessed spatial uncertainties due to aggregation from ecoregions to countries or the globe.

Sixth, the reference state should be interpreted as a “hypothetical biotic potential”. This also applies to our case, where the reference state is not the original or future successional biodiversity state at the same location as the land use but the current natural habitat elsewhere in the same ecoregion.

Seventh, alternative reference states should be tested. Like in the previous studies,^16,20,21^ we did not experiment with alternative reference states. This recommendation has the lowest priority, and we considered other types of sensitivity analyses more important.

Another source of uncertainty is the generation time required for land transformation impacts. Like previous studies,^16,20,21^ we used the estimates from Curran et al.^28^ They show large variations across five biogeographical realms. For example, passive restoration of plant species richness in forest biomes takes 50 to 100 years, depending on the realm. One can imagine that there are also differences among ecoregions within the same realm. Moreover, regeneration is assumed to be linear, whereas ecosystem dynamics are rather nonlinear.^58^

### Application

Interpretation of the resulting impact estimates has caused confusion in the past. It is important to emphasize that the CFs report potential and not actual fractions of species loss. An environmental pressure like land use might cause species to be lost locally or go extinct globally, but it might only happen with a delay, a phenomenon called the extinction debt.^59^ The longer the pressure persists, the more likely that the potential impacts occur, making the time dimension an important part of the unit. Occupation CFs are expressed in PDF/m^2^ and applied to inventory data reporting the area and time of occupation, whereas transformation CFs are expressed in PDF·yr/m^2^ and applied to the area of transformation. The time in the unit represents the regeneration time. Both result in impacts in PDF·yr.

CFs are available at the level of terrestrial ecoregions, countries, and the globe. We recommend using the ecoregion CFs whenever possible, as they constitute the native scale of the analysis, delineate boundaries that are meaningful for biodiversity, and offer more spatial detail by covering 825 ecoregions. Since inventory data, especially for background systems, are often available only at the country level, the country CFs facilitate the linking to such inventory data. We advise against using the globally aggregated CFs, given the importance of regionalization for biodiversity impacts.

CFs are available for both regional and global species loss. We generally recommend considering global species loss, as these impacts are irreversible, whereas regional species loss is reversible through species migration. However, regional species loss is more closely related to regional ecosystem functioning, which can also be of interest depending on the scope of the study. Since the ecoregion area influences the impacts to some extent, CFs for regional species loss can be considered to account for the scarcity of the affected ecosystem. Alternatively, the CFs could be multiplied by the ecoregion area, resulting in impacts in PDF·m^2^·yr, which was the common impact unit for local species loss in earlier LCIA indicators.^11^

Preferably, species groups are kept separately in the impact assessment to reflect their differences, but aggregation might be demanded for decision-making. Here, all species groups are given equal weight at the same taxonomic rank in the proposed aggregated CFs, i.e., first for animals and plants separately and then all together. Different approaches could be chosen, some of which are demonstrated by Verones et al.^51^

The application of average or marginal CFs depends on the scope of the study.^9,26^ For footprints of nations or large regions, for example, as in environmentally extended multiregional input-output analysis or in our global test application, average CFs are more suitable. In contrast, marginal CFs are more suitable for standard LCAs of products. The CFs are not representative of future scenarios with large additional land use for which new CFs would need to be developed. If such alternatives are lacking, then the marginal CFs would be more appropriate than average CFs but should be used with caution. Both average and marginal CFs can be used in attributional as well as consequential LCAs.^60^

The CFs consider the degree of land fragmentation within an ecoregion internally. This approach increases the ease of using CFs, as life cycle inventories typically do not report the degree of fragmentation. However, this implies that the CFs cannot be used to assess a change in the degree of fragmentation. Such an assessment would require both a different approach to considering fragmentation within the CFs and a more detailed life cycle inventory.

The CFs cover five land use types and three intensity levels. For impact assessment, they need to be linked to life cycle inventory data, which usually use a different land use classification. Scherer et al.^61^ provide guidance on such linking in general and for specific life cycle inventory databases. A difference to the linking they suggested applies to plantations. The linking was largely suggested based on the CFs from Chaudhary and Brooks,^20^ who limit plantations to timber plantations. Here, plantations also represent other types of plantations, such as oil palm and agroforestry.

Since not every land use type and at every intensity level occurs in each ecoregion, CFs could not be calculated for such cases. Still, there could be situations where CFs for these land uses are needed if hypothetical or prospective land use is expected to go beyond the present activities. Proxies were provided and flagged as such. However, it must be stressed that any use of such proxies must be done with caution.

Given the various sets of CFs provided, the user can choose a set aligned with the goal and scope of the study. The CFs are compatible with other CFs developed within GLAM3 regarding ecosystem quality, which all apply global extinction probabilities from Verones et al.^30^ Although uncertainties remain, the proposed CFs advance biodiversity impact assessment by using more recent data and considering multiple pressures: land use at different intensity levels and land fragmentation.

## Data Availability

The data underlying this study are openly available in Zenodo at https://doi.org/10.5281/zenodo.10114493.

## Supporting Information Available

Additional methods, tables related to input data and results, and figures, including a flowchart, numerous maps, as well as violin and scatter plots (PDF)
References of the data sets contributed to TRY that were used in the analysis (PDF)
