# Supplementary material for: Biodiversity Impact Assessment Considering Land Use Intensities and Fragmentation
Source: Environ Sci Technol. 2023 Nov 16;57(48):19612–23. doi: 10.1021/acs.est.3c04191 (PMC10702493; doi:10.1021/acs.est.3c04191)
Supplement: Supplementary file 1 — es3c04191_si_001.pdf [file es3c04191_si_001.pdf]

Supporting information 1

# Biodiversity Impact Assessment Considering Land Use Intensities and Fragmentation

*Laura Scherer[1,*], Francesca Rosa[2], Zhongxiao Sun[3], Ottar Michelsen[4], Valeria De Laurentiis[5], Alexandra Marques[6], Stephan Pfister[2], Francesca Verones[7], Koen J. J. Kuipers[8]*

[1] Institute of Environmental Sciences (CML), Leiden University, 2333 CC Leiden, The Netherlands

[2] Institute of Environmental Engineering, ETH Zurich, 8093 Zurich, Switzerland

[3] College of Land Science and Technology, China Agricultural University, Beijing 100083, China

[4] Department of Industrial Economics and Technology Management, Norwegian University of Science and Technology (NTNU), 7491 Trondheim, Norway

[5] European Commission-Joint Research Centre, 21027 Ispra, Italy

[6] PBL Netherlands Environmental Assessment Agency, 2500 GH The Hague, The Netherlands

[7] Industrial Ecology Programme, Department for Energy and Process Engineering, Norwegian University of Science and Technology (NTNU), 7491 Trondheim, Norway

[8] Department of Environmental Science, Radboud Institute for Biological and Environmental Sciences (RIBES), Radboud University, 6525AJ Nijmegen, The Netherlands

**Summary**

30 pages

30 figures

7 tables

## **Supporting methods**

Characterization factor framework

The probability of dispersal is based on the least-cost distance ($w$, m) between patches $x$ and $y$ and the median dispersal distance ($\alpha$, m) of species group $g$ in region $j$ (eq. S1).[1]

$$p_{g,j,i,m,x,y} = e^{-w_{g,j,i,m,x,y}/\alpha_{g,j,i}} \text{ (Eq. S1)}$$

The least-cost distance is determined by the interpatch distance ($d$, m) and the resistance ($r$, dimensionless) of the surrounding land type $k$ separating the patches (eq. S2).

$$w_{g,j,i,m,x,y} = min\left(\sum_k d_{j,i,m,x,m} \cdot r_{g,j,i,m,k}\right) \text{ (Eq. S2)}$$

The allocation factor ($a$, dimensionless) is based on the area ($A$, $m^2$) and the habitat affinity ($h$, dimensionless) of the land type $i$ (here excluding primary vegetation) and use *intensity m* (eq. S3).[2]

$$a_{g,j,i,m} = \frac{\left(1-h_{g,j,i,m}{}^{z_{g,j}}\right)\cdot A_{j,i,m}}{\sum_{i,m}\left(1-h_{g,j,i,m}{}^{z_{g,j}}\right)\cdot A_{j,i,m}} \text{ (Eq. S3)}$$

Model parametrization for plants

In the raw data of Gallego-Zamorano et al.[3], we changed the classification of urban forests from intense to light use. Moreover, we removed six data records (one urban, two cropland, and three plantation forests), as they were identified as outliers based on the modified z-score, leaving 319 data records for further analysis. While Gallego-Zamorano et al.[3] only considered three intensity levels of cropland and two of pasture and otherwise only considered broad land use types, we distinguished more often three intensity levels, including pasture and urban areas. We considered primary and secondary vegetation with the vegetation type forest as managed forest. Primary vegetation with a minimal use intensity served as the reference land use. If the change in species richness relative to the reference land use exceeded 1, which happened for minimally used urban land, it was cut off at 1 to avoid giving benefits under the high uncertainty of the underlying data.

A z-value for the biome "mangroves" was missing and assumed to equal flooded grasslands and savannas, as both are water-inundated and have equal z-values for mammals.[4]

To link the species with traits to those with spatial distributions, the names in both databases were standardized based on the World Flora Online taxonomic backbone[5] and exact and fuzzy matching.[6] If only the genus could be matched, we replaced this part in the species name and tried to match it again. If the fuzzy distance was larger than 2 or equal to 2 but both changes were substitutions as opposed to insertions or deletions, we considered the matching too imprecise and the species unmatched. Following this name standardization, some names turned out to be synonyms, and duplicates were removed from the plant species distributions, leaving 26,573 vascular plant species for further analysis.

The trait categories of the "dispersal syndrome" and "growth form" needed to be harmonized and aligned with the categories of the linear regression model. The dispersal syndrome could include the categories animal (vertebrate), ant, ballistic, wind (none, i.e. without special adaptation), and wind (special, i.e., with the help of seed appendages). Since TRY did not allow for a distinction between different forms of wind dispersal, we assumed all to be wind

(special), as this category was more common in the dataset of Tamme et al.[7]. The growth form included the categories tree, shrub, and herb. If TRY provided multiple values for a certain species and trait, we would select the most frequent value.

Model parametrization for vertebrates

We received the dataset used for Newbold et al.[8] and the demo script to obtain the relative species richness per land use class via private communication with Tim Newbold (July 2022). The demo script used the functions CorrectSamplingEffort, MergeSites and SiteMetrics from the library predictsFunctions of Newbold's GitHub repository.[9] We adapted the script so that we could include some confounding variables when modelling the species richness response to land use (as done in the original publication): the distance to road,[10] the population density[11] and the estimated travel time to the nearest city of 50'000 or more people.[12] Subsequent to using the GLMER function, we used the function PlotGLMERFactor,[9] which we adapted to retrieve the species richness difference expressed as percentage between natural habitat and the land use intensity levels resulting from the model. This way, we could obtain the relative species richness per land use intensity level by dividing the species richness difference expressed as a percentage by 100 and adding 1.

The data from the meta-analysis[13] are specific to the forest management category and species group at continental resolution (the raw data can be found in the supporting information of the reference, Excel sheet *Raw data*, columns *Xc* and *Xe*). *Xe* represents the "mean species richness in disturbed (managed) forest sites", and *Xc* represents the "mean species richness in reference (unmanaged) forest sites". For intense management, we used the data on clear-cut (all trees in the harvest areas are removed at once, resulting in even-aged silviculture); for light management, we used the data on selective logging (largest, highest and oldest quality trees are removed, while the remaining vegetation is left standing); for minimum management, we used the data on reduced impact logging (similar to selective logging, but adopted with more sustainable practices, e.g., to reduce the soil damage, biodiversity impact, etc.).

As for plants, a cut-off at 1 was adopted when the response ratios were above 1.

Since the habitat affinities of vertebrates depend on the species numbers (eq. 9), the characterization factors for ecoregions with only one species recorded were considered unreliable and set to a missing value.

Model parametrization regarding land use and intensity

Regarding cropland, we used the cropland from HILDA+[14] as the base map and the area equipped for irrigation[15] and phosphorus and nitrogen fertilizer use[16] as supplementary maps. The fertilizer use maps were disaggregated from 0.5° to 5 arcminutes by assigning the value of the original larger cell to all smaller cells within it. If the area equipped for irrigation and both fertilizer uses did not exceed the first quartile of non-zero values, it would be considered minimal use. If the area equipped for irrigation and fertilizer uses were below or equal to the third quartile, the cropland would be considered light use. If the area equipped for irrigation or any of the fertilizer uses exceeded the third quartile, the cropland would be considered intense use.

Regarding pasture, we used the pasture/rangeland from HILDA+ as the base map and land use from GLOBIO 4[17] as a supplementary map. We considered “grassland” from GLOBIO 4 as minimal use, “rangeland” as light use, and “pasture” as intense use. If there was a mismatch between the broad land use types of HILDA+ and GLOBIO 4, we also assumed light use. The GLOBIO 4 data was first reclassified to assign numeric values to the three intensity levels and then averaged to align the resolution with the base map.

Regarding plantations, we used the forest from HILDA+ as the base map and forest management[18] and oil palm plantation data[19] as supplementary maps. We considered “agroforestry” among the forest management classes as minimal use, “planted forests” among the forest management classes and smallholder oil palm plantations as light use, and “plantation forests” among the forest management classes and industrial oil palm plantations as intense use. The oil palm plantation data were first aggregated based on the most frequent category from 10 to 100 m to match the resolution of the forest management data, both were reclassified to the three intensity levels, and then averaged to align the resolution again with the base map.

Regarding managed forests, we used the forest from HILDA+ again as the base map but removed natural forests and plantations. This mainly left the type “naturally regenerating forests with signs of forest management, e.g., logging, clear cuts etc.”,[18] as well as those cells for which no forest management class was indicated. Within the remaining forest areas, we estimated the share of 30-m cells with forest in 2000 that experienced forest extent loss in 2020 within 5-arcmin cells based on data from Potapov et al.[20]. We considered forest extent loss below or equal to 2% as minimal use, between 2% and 20% as light use, and above 20% as intense use. The resulting share in grid cells with minimal use is similar to the one for the classes from the Global Land Systems[21] that Newbold et al.[8] linked to primary and secondary vegetation with minimal use and that are relevant to forests. We included natural forests in this comparison, as Newbold et al.[8] do not distinguish between natural and managed forests. Our share for light use is lower and for intense use higher. This seems justified because 1) the Global Land Systems only allowed considering livestock for the definition of intensity levels, while also other factors can contribute to light or intense use of managed forests, 2) their map represents the year 2000, while our CFs represent the year 2015, and there is a general trend towards more intensification, and 3) following a precautionary principle, overestimating the intensity level would be preferred over underestimating it.

Regarding urban areas, we used the urban area from HILDA+ as the base map and the Global Human Settlement[22] as a supplementary map. We considered the categories “Very low density rural”, “Low density rural”, and “rural cluster” as minimal use, “suburban or peri-urban” and “semi-dense urban cluster” as light use, and “dense urban cluster” and “urban cluster” as intense use. Again, we averaged the reclassified intensity levels for aggregation.

## Supporting tables

Table S1. Relative species richness of plants and vertebrates. N: sample size, RR: relative species richness, lb and ub: lower and upper bounds of the 95% confidence interval.

| Land use type and intensity | N | RR | RR_lb | RR_ub |
|---|---|---|---|---|
| **Plants** | | | | |
| Primary_vegetation_Minimal | 86 | 1.00 | 0.92 | 1.10 |
| Cropland_Intense | 8 | 0.22 | 0.17 | 0.29 |
| Cropland_Light | 7 | 0.48 | 0.37 | 0.64 |
| Cropland_Minimal | 11 | 0.54 | 0.43 | 0.68 |
| Managed_forest_Intense | 8 | 0.49 | 0.38 | 0.64 |
| Managed_forest_Light | 18 | 0.71 | 0.59 | 0.85 |
| Managed_forest_Minimal | 94 | 0.91 | 0.83 | 1.00 |
| Pasture_Intense | 6 | 0.48 | 0.35 | 0.65 |
| Pasture_Light | 6 | 0.64 | 0.48 | 0.87 |
| Pasture_Minimal | 7 | 0.93 | 0.70 | 1.23 |
| Plantation_Intense | 7 | 0.29 | 0.22 | 0.39 |
| Plantation_MinimalLight | 43 | 0.59 | 0.51 | 0.67 |
| Urban_Intense | 3 | 0.44 | 0.29 | 0.68 |
| Urban_Light | 5 | 0.98 | 0.71 | 1.37 |
| Urban_Minimal | 4 | 1.46* | 1.01 | 2.11 |
| **Vertebrates** | | | | |
| Primary_vegetation_Minimal | 2515 | 1.00 | 1.00 | 1.00 |
| Cropland_LightIntense | 1488 | 0.69 | 0.61 | 0.79 |
| Cropland_Minimal | 505 | 0.84 | 0.74 | 0.96 |
| Managed_forest_Intense | 41 | 0.67 | 0.31 | 1.00 |
| Managed_forest_Light | 125 | 0.91 | 0.38 | 1.00 |
| Managed_forest_Minimal | 16 | 0.98 | 0.73 | 1.00 |
| Pasture_LightIntense | 1004 | 0.75 | 0.67 | 0.83 |
| Pasture_Minimal | 807 | 0.85 | 0.77 | 0.95 |
| Plantation_LightIntense | 1293 | 0.79 | 0.72 | 0.87 |
| Plantation_Minimal | 336 | 0.98 | 0.89 | 1.08 |
| Urban_Intense | 111 | 0.69 | 0.54 | 0.87 |
| Urban_Light | 364 | 0.83 | 0.69 | 1.00 |
| Urban_Minimal | 226 | 1.06* | 0.91 | 1.24 |

* Values > 1 were set to 1 for further analyses.

Table S2. Land use classification by broad type and intensity, following Extended Data Table 1 from Newbold et al.[8].

| Type | Intensity | Details |
|---|---|---|
| Primary and secondary vegetation (used as a basis for managed forests) | Minimal | Any disturbances identified are very minor (e.g., a trail or path) or very limited in the scope of their effect (e.g., hunting of a particular species of limited ecological importance). |
| | Light | One or more disturbances of moderate intensity (e.g., selective logging) or breadth of impact (e.g., bushmeat extraction), which are not severe enough to markedly change the nature of the ecosystem. Primary sites in suburban settings are at least Light use. |

| | | |
|---|---|---|
| | Intense | One or more disturbances that are severe enough to markedly change the nature of the ecosystem; this includes clear-felling of part of the site too recently for much recovery to have occurred. Primary sites in fully urban settings should be classed as Intense use. |
| Plantation forests (called plantations in our study) | Minimal | Extensively managed or mixed timber, fruit/coffee, oil-palm or rubber plantations in which native understorey and/or other native tree species are tolerated, which are not treated with pesticide or fertilizer, and which have not been recently (< 20 years) clear-felled. |
| | Light | Monoculture fruit/coffee/rubber plantations with limited pesticide input, or mixed species plantations with significant inputs. Monoculture timber plantations of mixed age with no recent (< 20 years) clear-felling. Monoculture oil palm plantations with no recent (< 20 years) clear-felling. |
| | Intense | Monoculture fruit/coffee/rubber plantations with significant pesticide input. Monoculture timber plantations with similarly aged trees or timber/oil palm plantations with extensive recent (< 20 years) clear-felling. |
| Pasture | Minimal | Pasture with minimal input of fertilizer and pesticide, and with low stock density (*not* high enough to cause significant disturbance or to stop regeneration of vegetation). |
| | Light | Pasture either with significant input of fertilizer or pesticide, or with high stock density (high enough to cause significant disturbance or to stop regeneration of vegetation). |
| | Intense | Pasture with significant input of fertilizer or pesticide, *and* with high stock density (high enough to cause significant disturbance or to stop regeneration of vegetation). |
| Cropland | Minimal | Low-intensity farms, typically with small fields, mixed crops, crop rotation, little or no inorganic fertilizer use, little or no pesticide use, little or no ploughing, little or no irrigation, little or no mechanization. |
| | Light | Medium-intensity farming, typically showing some but not many of the following: large fields, annual ploughing, inorganic fertilizer application, pesticide application, irrigation, no crop rotation, mechanization, monoculture crop. Organic farms in developed countries often fall within this category, as may high-intensity farming in developing countries. |
| | Intense | High-intensity monoculture farming, typically showing many of the following features: large fields, annual ploughing, inorganic fertilizer application, pesticide application, irrigation, mechanization, no crop rotation. |
| Urban | Minimal | Extensive managed green spaces; villages. |
| | Light | Suburban (e.g., gardens), or small managed or unmanaged green spaces in cities. |
| | Intense | Fully urban with no significant green spaces. |

Table S3. Application of average characterization factors to global land occupation in 2015. All characterization factors used in this application are aggregated across the species groups and represent relative global species losses.

| **Spatial level of characterization factors** | **Impact (PDF·yr)** |
|---|---|
| Terrestrial ecoregions (native scale) | 0.156 |
| Countries | 0.178 |
| Globe (weighted by land use) | 0.156 |
| Globe (weighted by ecoregion area, only areas with current land use) | 0.183 |

| | |
|---|---|
| Globe (weighted by ecoregion area, considering proxies) | 0.326 |

Table S4. Comparison with other CFs and IUCN data. r is the Spearman correlation coefficient, pbias is the percent bias, and $n_{threat}$ is the number of species threatened by land use. Subscript 21 refers to Kuipers et al. (2021)[23], and 18 to Chaudhary and Brooks (2018)[24]. The correlation between the CFs from this study and those from GLAM1 is 0.79 for global, taxa-aggregated CFs, and the percent bias is -50. See also Figure S29 and Figure S30.

| | **$r_{21}$** | **$pbias_{21}$** | **$r_{18}$** | **$pbias_{18}$** | **$r_{18\text{-}21}$** | **$pbias_{18\text{-}21}$** | **$n_{threat,IUCN}$** | **$n_{threat}$** |
|---|---|---|---|---|---|---|---|---|
| Plants | - | - | 0.74 | -22 | - | - | 4175 | 1741 |
| Amphibians | 0.64 | -46 | 0.83 | 7 | 0.68 | 54 | 1865 | 214 |
| Birds | 0.71 | -12 | 0.87 | 104 | 0.75 | 80 | 1131 | 611 |
| Mammals | 0.74 | -30 | 0.80 | 76 | 0.70 | 111 | 819 | 289 |
| Reptiles | 0.71 | -39 | 0.84 | -34 | 0.70 | -34 | 752 | 318 |

Table S5. Sensitivity analysis. r is the Spearman correlation coefficient, and pbias is the percent bias. Subscripts lb and ub refer to lower and upper bounds for the local relative species richness, broad to broad land use types without distinguishing land use intensities, and sar to the species-area relationship without considering fragmentation.

| | **$r_{lb}$** | **$pbias_{lb}$** | **$r_{ub}$** | **$pbias_{ub}$** | **$r_{broad}$** | **$pbias_{broad}$** | **$r_{sar}$** | **$pbias_{sar}$** |
|---|---|---|---|---|---|---|---|---|
| Plants | 0.98 | 20 | 0.95 | -29 | 0.96 | 0.2 | 0.90 | -38 |
| Amphibians | 0.93 | 19 | 0.99 | -5 | 0.99 | -2 | 0.92 | -37 |
| Birds | 0.89 | 33 | 0.97 | -10 | 0.98 | 6 | 0.93 | -21 |
| Mammals | 0.91 | 29 | 0.96 | -12 | 0.99 | 4 | 0.93 | -30 |
| Reptiles | 0.93 | 19 | 0.99 | -5 | 0.99 | -0.3 | 0.92 | -36 |

Table S6. Contribution to variance (%). Analysis performed for global, average characterization factors for land occupation.

| | **Amphibians** | **Birds** | **Mammals** | **Plants** | **Reptiles** | **Average** |
|---|---|---|---|---|---|---|
| Habitat affinity (h) | 21.7 | 57.0 | 59.7 | 33.7 | 19.7 | 38.4 |
| Equivalent connected area (ECA) relative to area used | 2.5 | 2.1 | 2.4 | 2.7 | 0.1 | 2.0 |
| Area used related to ecoregion area | 0.4 | 0.5 | 0.6 | 0.1 | 0.9 | 0.5 |
| Ecoregion area | 7.7 | 7.0 | 5.4 | 11.6 | 13.1 | 9.0 |
| z-value | 9.7 | 9.2 | 6.0 | 15.5 | 12.8 | 10.6 |
| Global extinction probability (GEP) | 58.0 | 24.2 | 25.9 | 36.4 | 53.4 | 39.6 |

Table S7. Correlation matrix of global, average, land occupation characterization factors across species groups.

| | **Plants** | **Amphibians** | **Birds** | **Mammals** |
|---|---|---|---|---|
| **Amphibians** | 0.38 | - | - | - |
| **Birds** | 0.21 | 0.78 | - | - |
| **Mammals** | 0.21 | 0.79 | 0.87 | - |
| **Reptiles** | 0.27 | 0.79 | 0.80 | 0.81 |

## Supporting figures

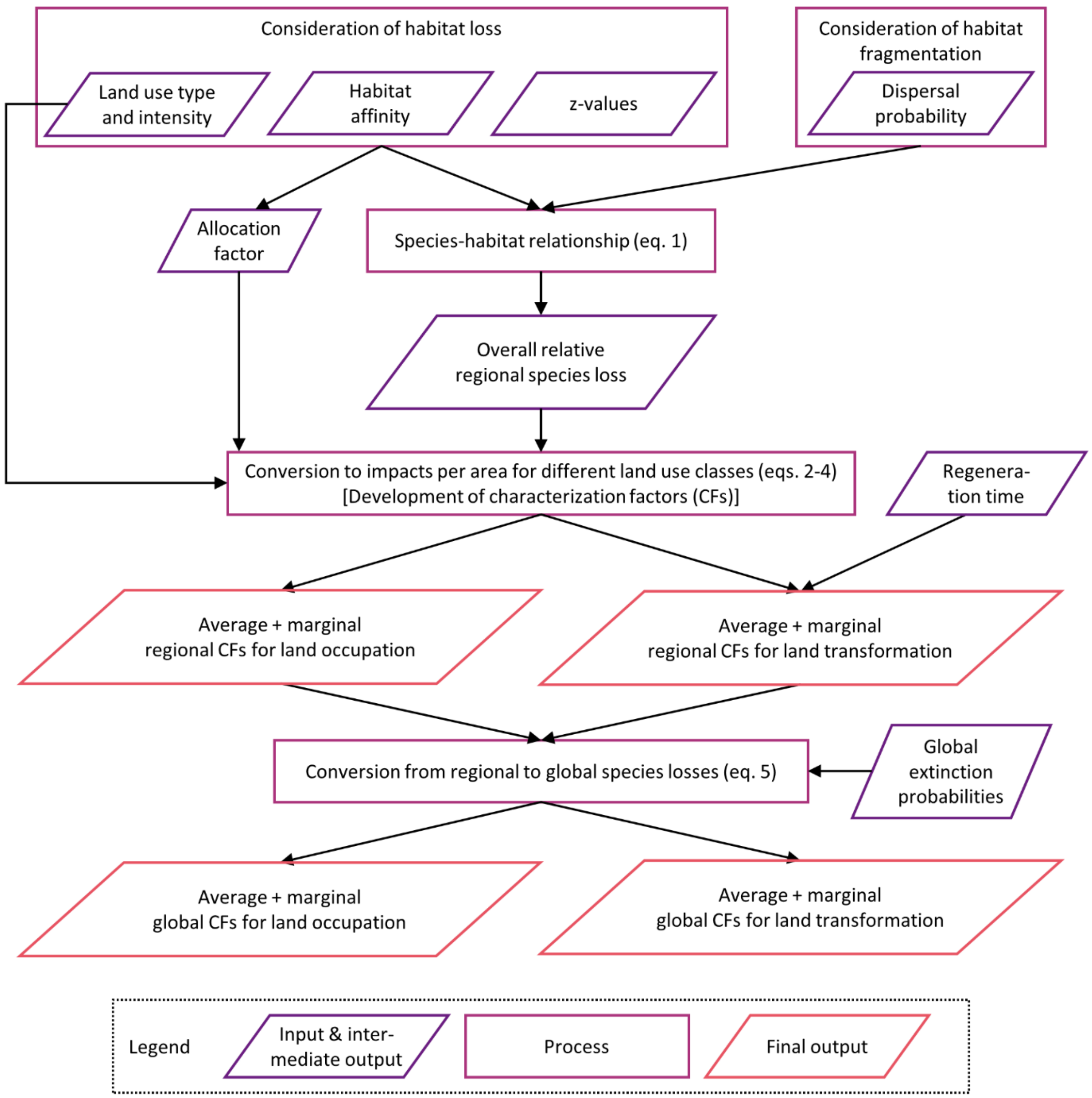


Figure S1. Conceptual overview of the methodological steps and main inputs necessary to produce the characterization factors (CFs).

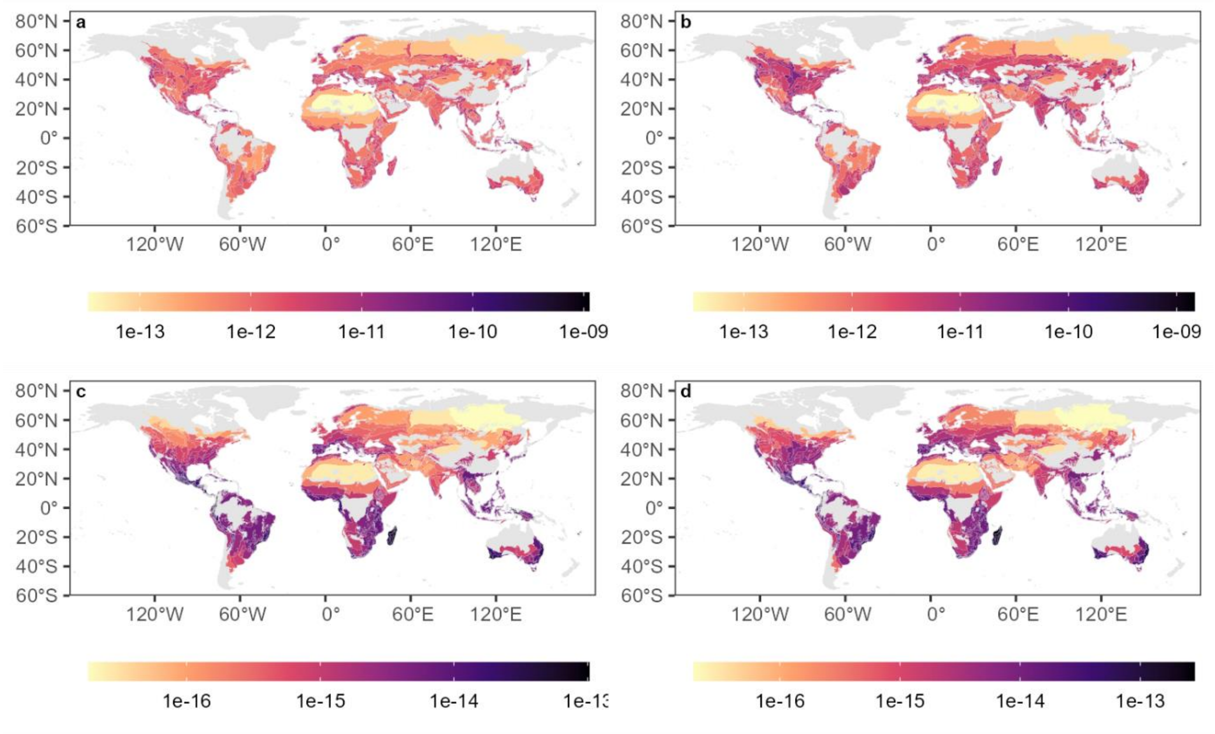


Figure S2. Land occupation characterization factors at the ecoregion level for cropland with light use and potential impacts on plant species richness. The unit is PDF/m$^2$. a) average impacts on regional species richness, b) marginal impacts on regional species richness, c) average impacts on global species richness, d) marginal impacts on global species richness. Grey denotes no data, indicating either the absence of the specific land use class in these regions or missing species data. More characterization factors are available through the use of proxies.

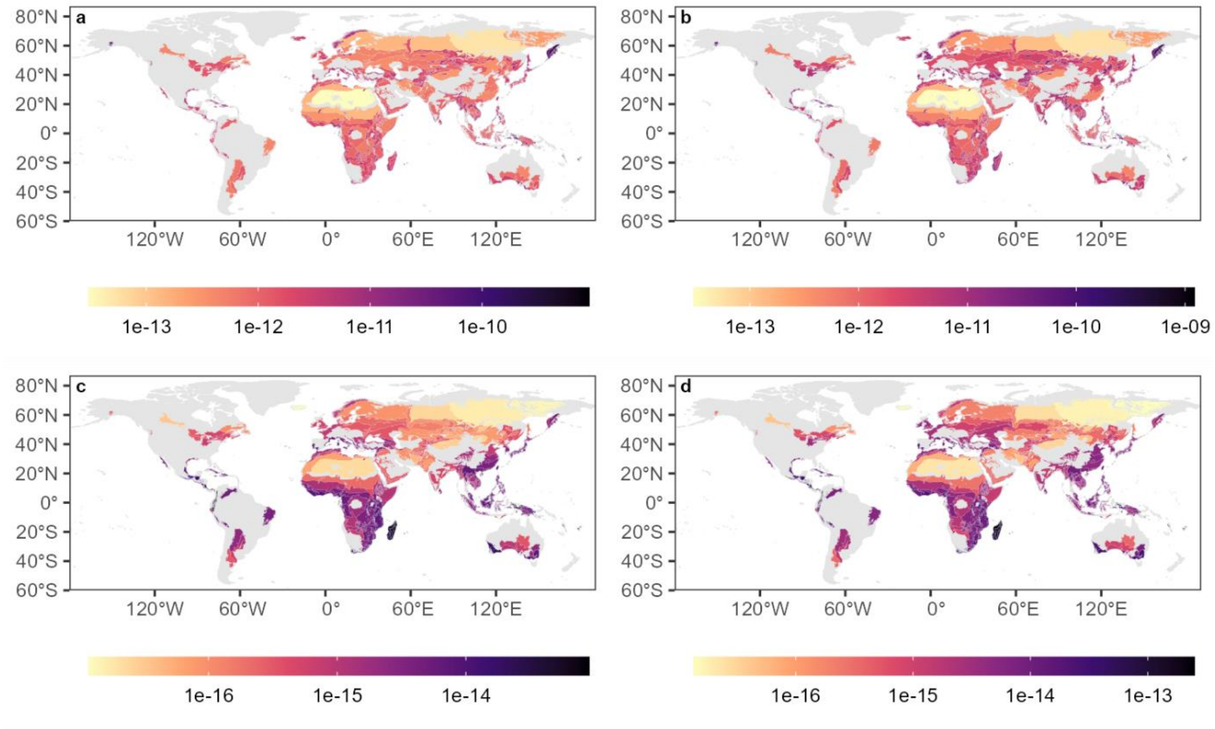


Figure S3. Land occupation characterization factors at the ecoregion level for cropland with minimal use and potential impacts on plant species richness. The unit is PDF/m$^2$. a) average impacts on regional species richness, b) marginal impacts on regional species richness, c) average impacts on global species richness, d) marginal impacts on global species richness. Grey denotes no data, indicating either the absence of the specific land use

class in these regions or missing species data. More characterization factors are available through the use of proxies.

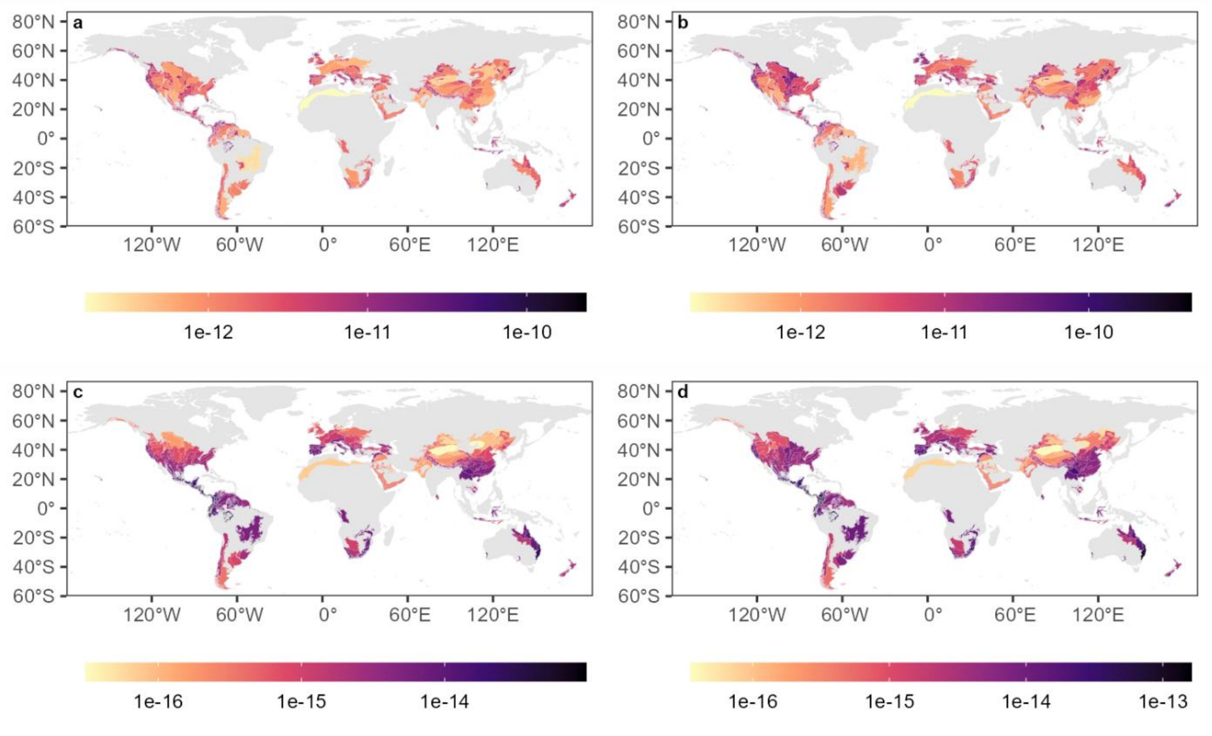


Figure S4. Land occupation characterization factors at the ecoregion level for pasture with intense use and potential impacts on plant species richness. The unit is PDF/$m^2$. a) average impacts on regional species richness, b) marginal impacts on regional species richness, c) average impacts on global species richness, d) marginal impacts on global species richness. Grey denotes no data, indicating either the absence of the specific land use class in these regions or missing species data. More characterization factors are available through the use of proxies.

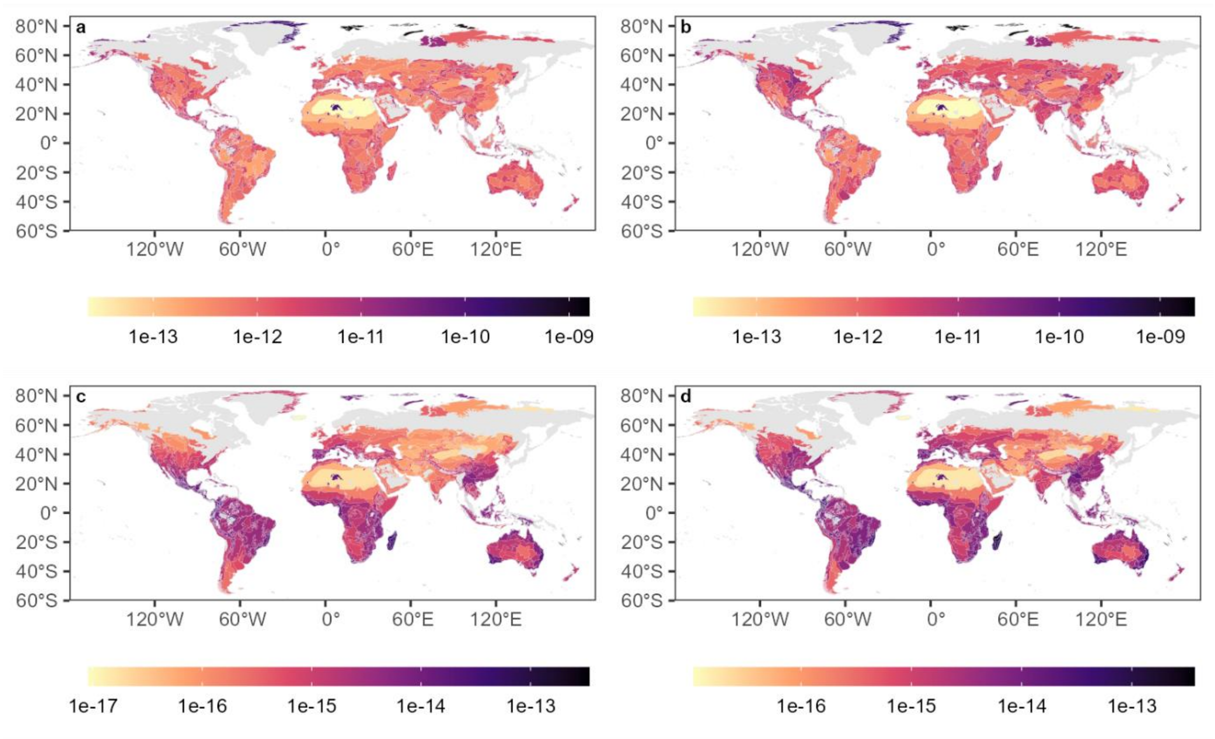


Figure S5. Land occupation characterization factors at the ecoregion level for pasture with light use and potential impacts on plant species richness. The unit is PDF/m$^2$. a) average impacts on regional species richness, b) marginal impacts on regional species richness, c) average impacts on global species richness, d) marginal impacts on global species richness. Grey denotes no data, indicating either the absence of the specific land use class in these regions or missing species data. More characterization factors are available through the use of proxies.

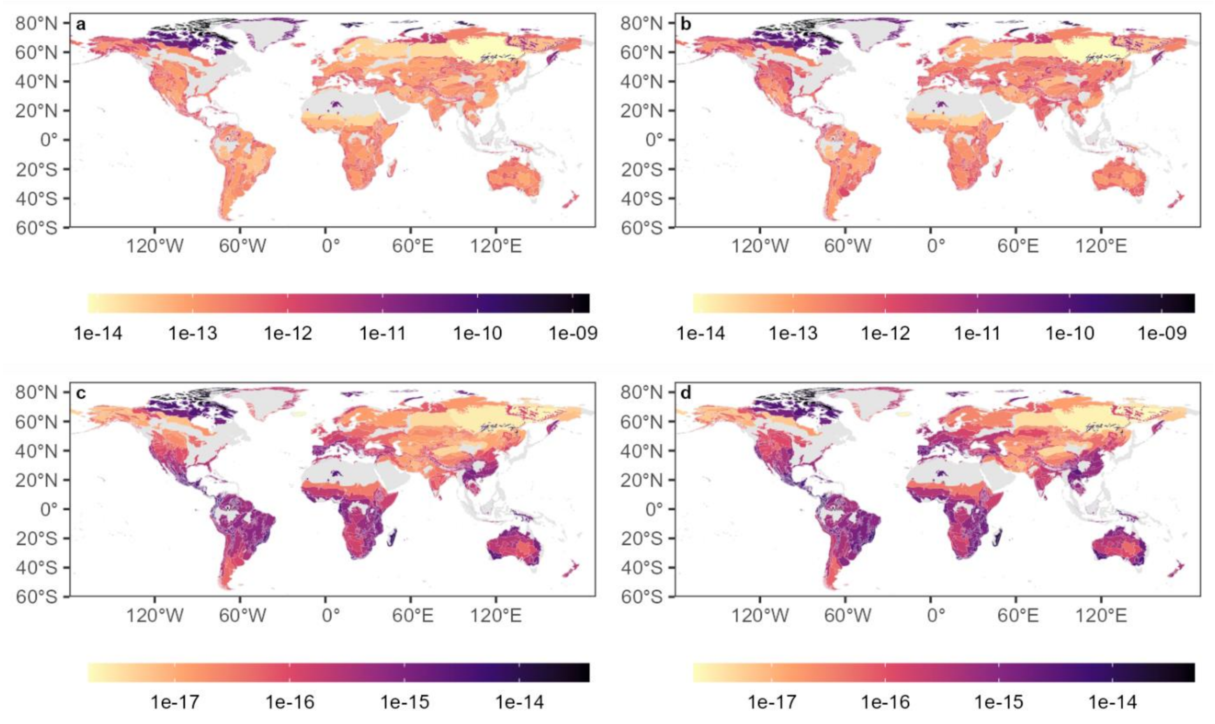


Figure S6. Land occupation characterization factors at the ecoregion level for pasture with minimal use and potential impacts on plant species richness. The unit is PDF/m$^2$. a) average impacts on regional species richness, b) marginal impacts on regional species richness, c) average impacts on global species richness, d) marginal

impacts on global species richness. Grey denotes no data, indicating either the absence of the specific land use class in these regions or missing species data. More characterization factors are available through the use of proxies.

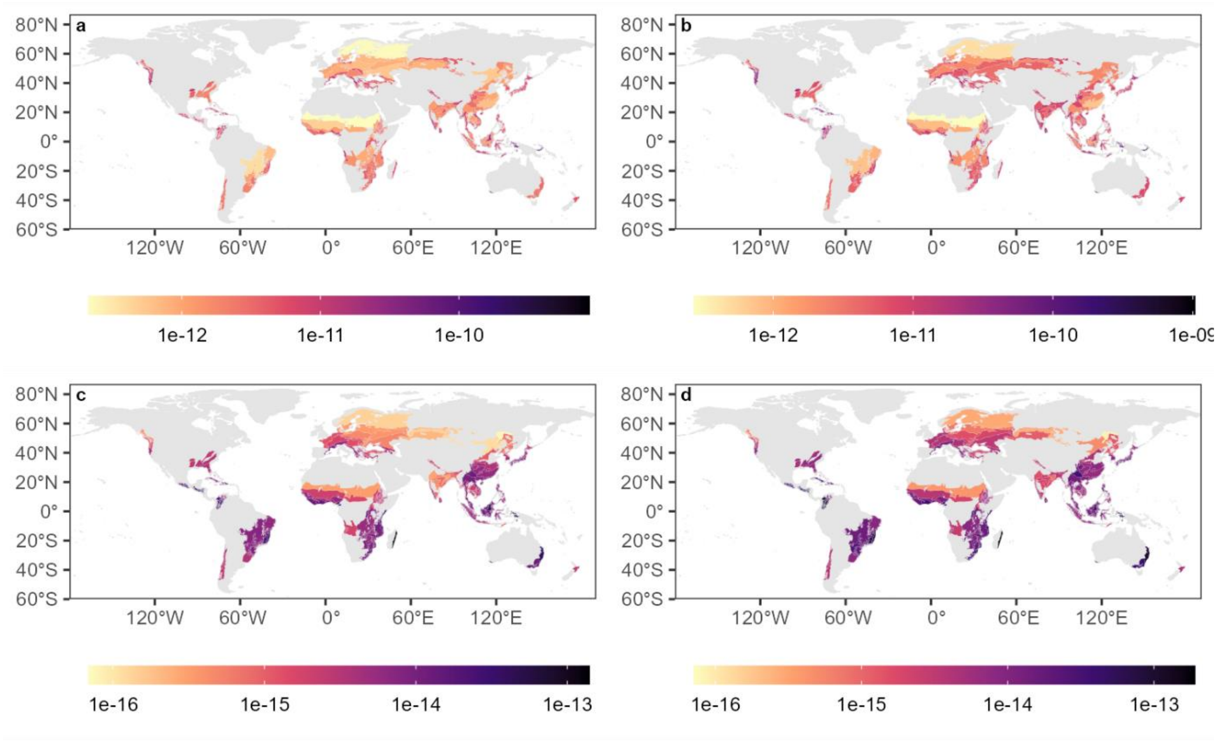


Figure S7. Land occupation characterization factors at the ecoregion level for plantations with intense use and potential impacts on plant species richness. The unit is PDF/m$^2$. a) average impacts on regional species richness, b) marginal impacts on regional species richness, c) average impacts on global species richness, d) marginal impacts on global species richness. Grey denotes no data, indicating either the absence of the specific land use class in these regions or missing species data. More characterization factors are available through the use of proxies.

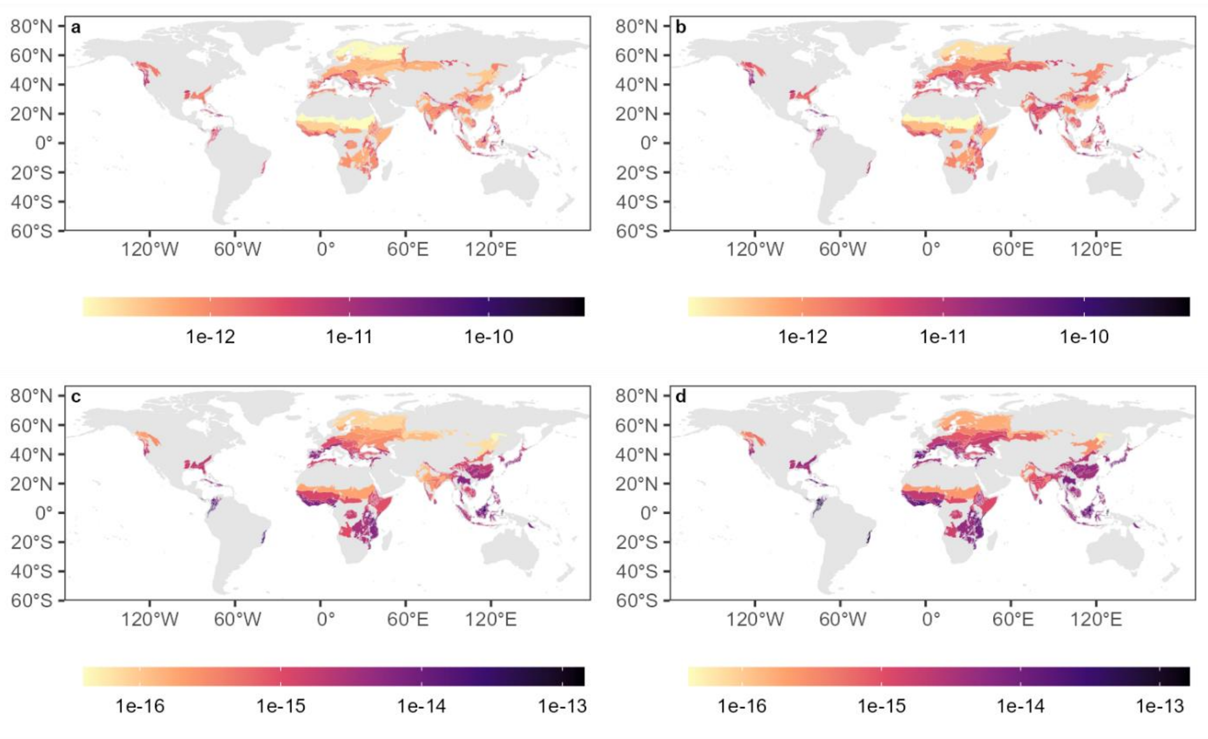


Figure S8. Land occupation characterization factors at the ecoregion level for plantations with light or minimal use and potential impacts on plant species richness. The unit is PDF/m$^2$. a) average impacts on regional species richness, b) marginal impacts on regional species richness, c) average impacts on global species richness, d) marginal impacts on global species richness. Grey denotes no data, indicating either the absence of the specific land use class in these regions or missing species data. Note that the impacts of plantations with light or minimal use on plant species richness are not differentiated. More characterization factors are available through the use of proxies.

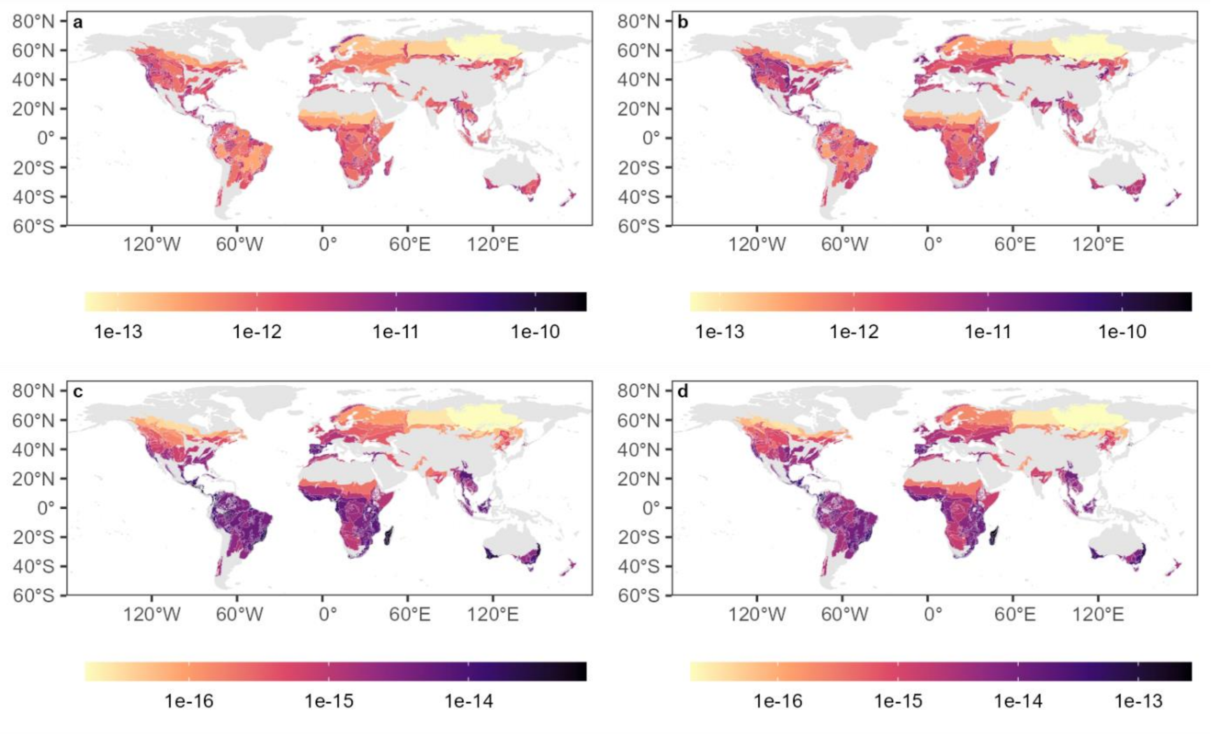


Figure S9. Land occupation characterization factors at the ecoregion level for managed forest with intense use and potential impacts on plant species richness. The unit is PDF/$m^2$. a) average impacts on regional species richness, b) marginal impacts on regional species richness, c) average impacts on global species richness, d) marginal impacts on global species richness. Grey denotes no data, indicating either the absence of the specific land use class in these regions or missing species data. More characterization factors are available through the use of proxies.

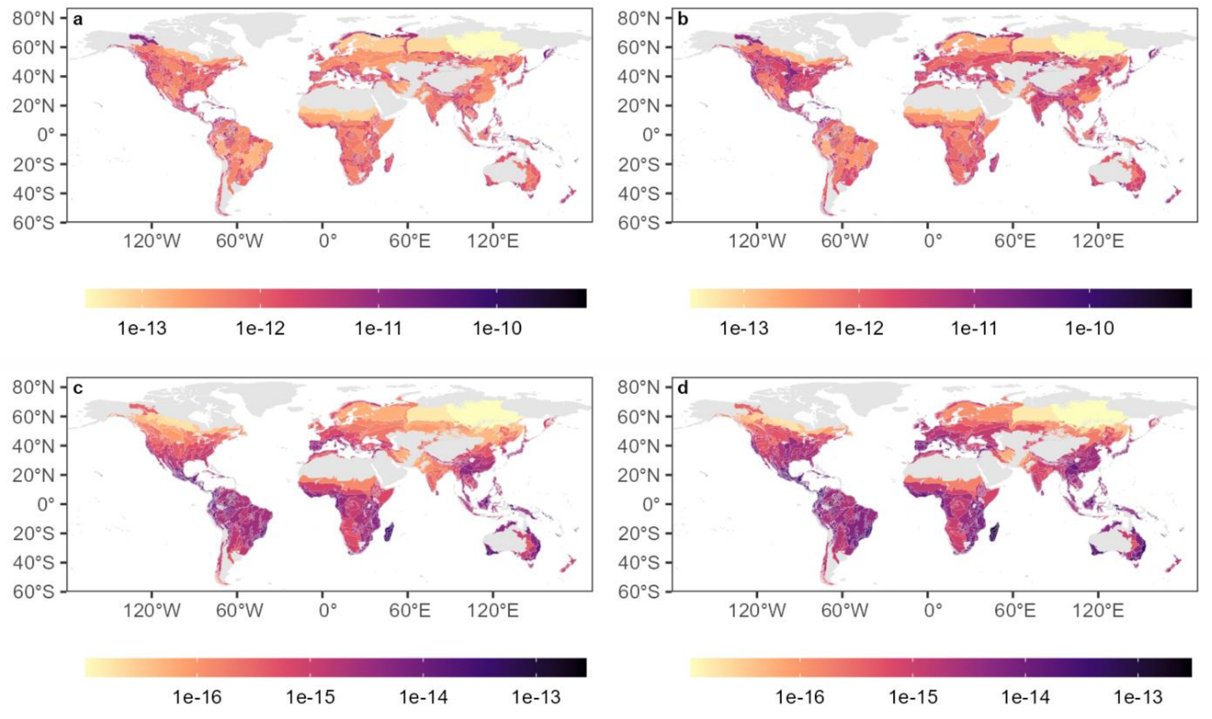


Figure S10. Land occupation characterization factors at the ecoregion level for managed forest with light use and potential impacts on plant species richness. The unit is PDF/$m^2$. a) average impacts on regional species richness, b) marginal impacts on regional species richness, c) average impacts on global species richness, d) marginal

impacts on global species richness. Grey denotes no data, indicating either the absence of the specific land use class in these regions or missing species data. More characterization factors are available through the use of proxies.

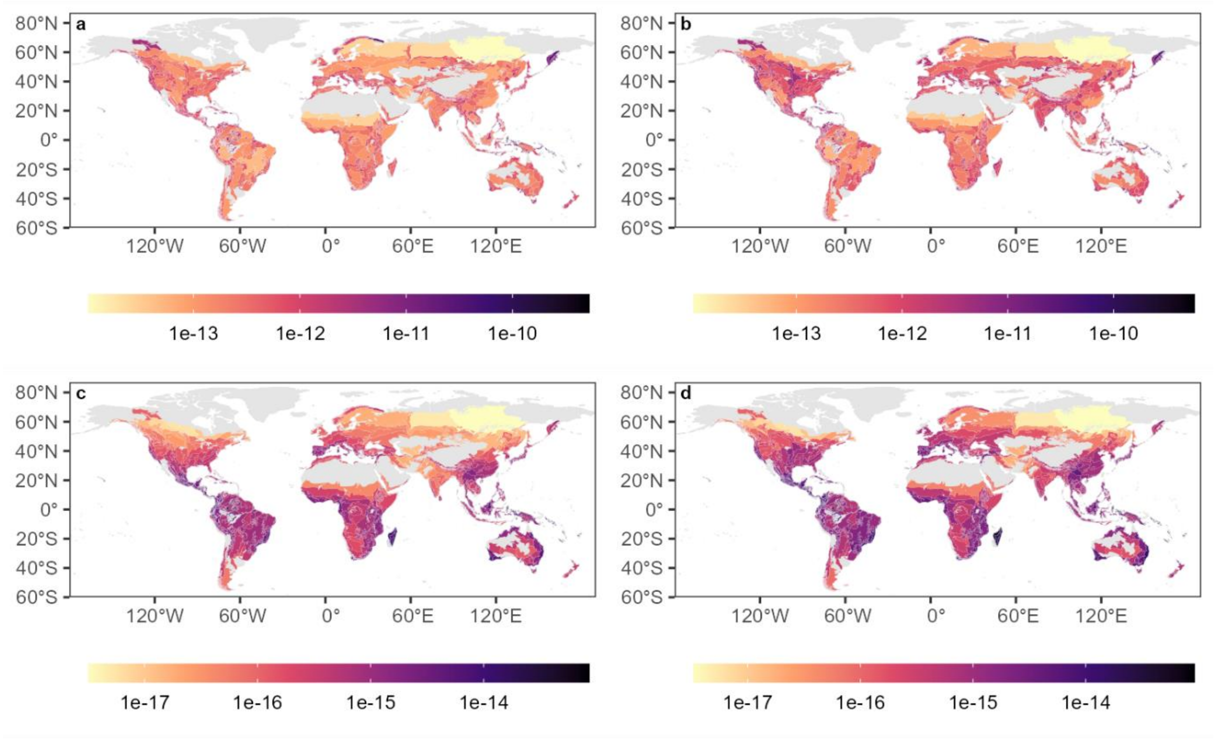


Figure S11. Land occupation characterization factors at the ecoregion level for managed forest with minimal use and potential impacts on plant species richness. The unit is PDF/m$^2$. a) average impacts on regional species richness, b) marginal impacts on regional species richness, c) average impacts on global species richness, d) marginal impacts on global species richness. Grey denotes no data, indicating either the absence of the specific land use class in these regions or missing species data. More characterization factors are available through the use of proxies.

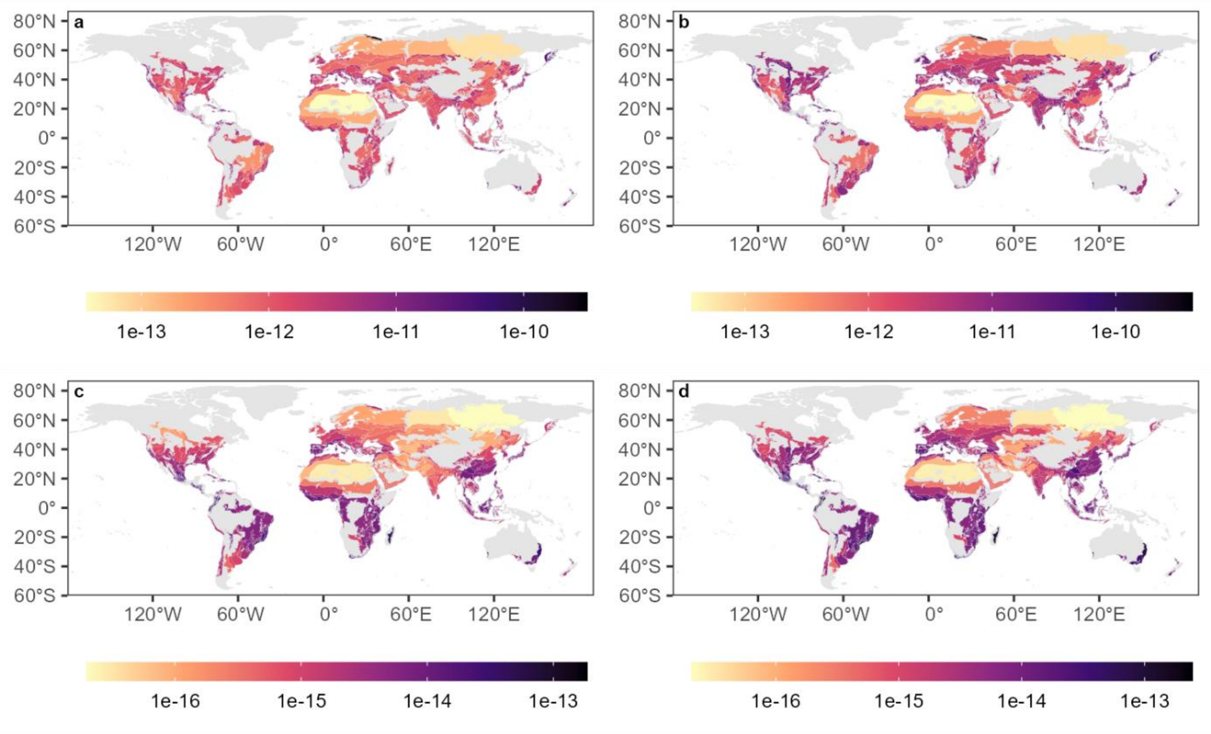


Figure S12. Land occupation characterization factors at the ecoregion level for urban areas with intense use and potential impacts on plant species richness. The unit is PDF/m$^2$. a) average impacts on regional species richness, b) marginal impacts on regional species richness, c) average impacts on global species richness, d) marginal impacts on global species richness. Grey denotes no data, indicating either the absence of the specific land use class in these regions or missing species data. More characterization factors are available through the use of proxies.

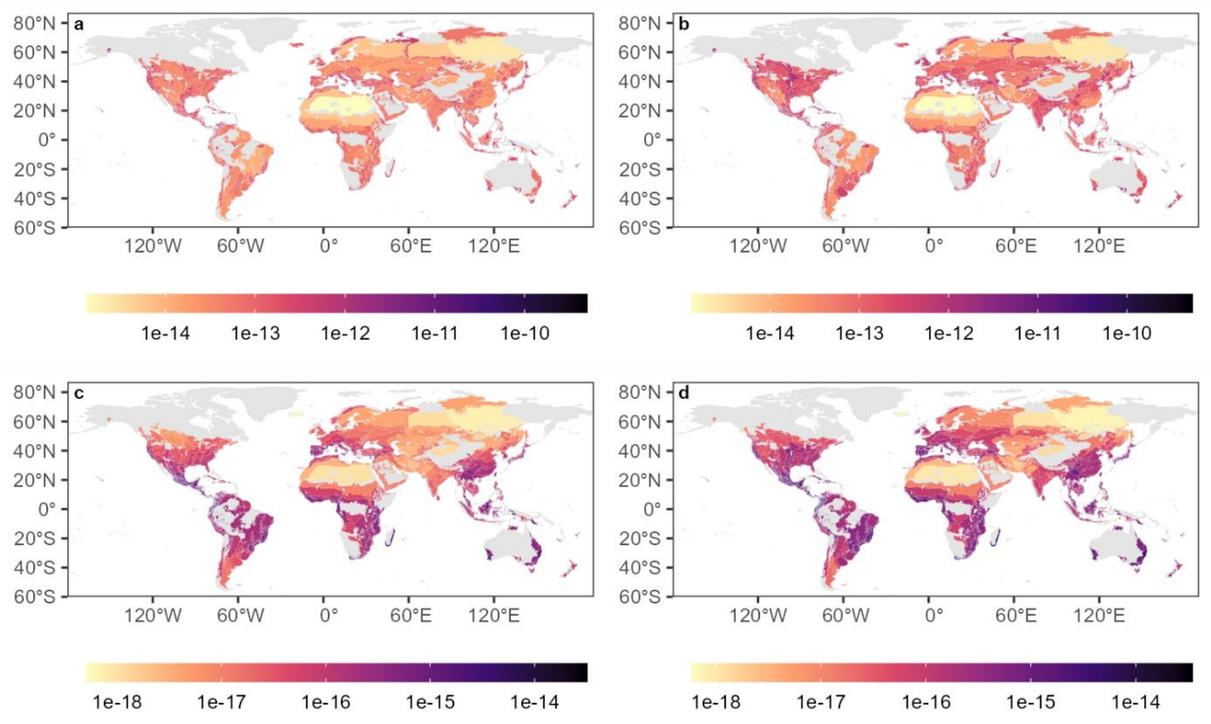


Figure S13. Land occupation characterization factors at the ecoregion level for urban areas with light use and potential impacts on plant species richness. The unit is PDF/m$^2$. a) average impacts on regional species richness, b) marginal impacts on regional species richness, c) average impacts on global species richness, d) marginal

impacts on global species richness. Grey denotes no data, indicating either the absence of the specific land use class in these regions or missing species data. More characterization factors are available through the use of proxies.

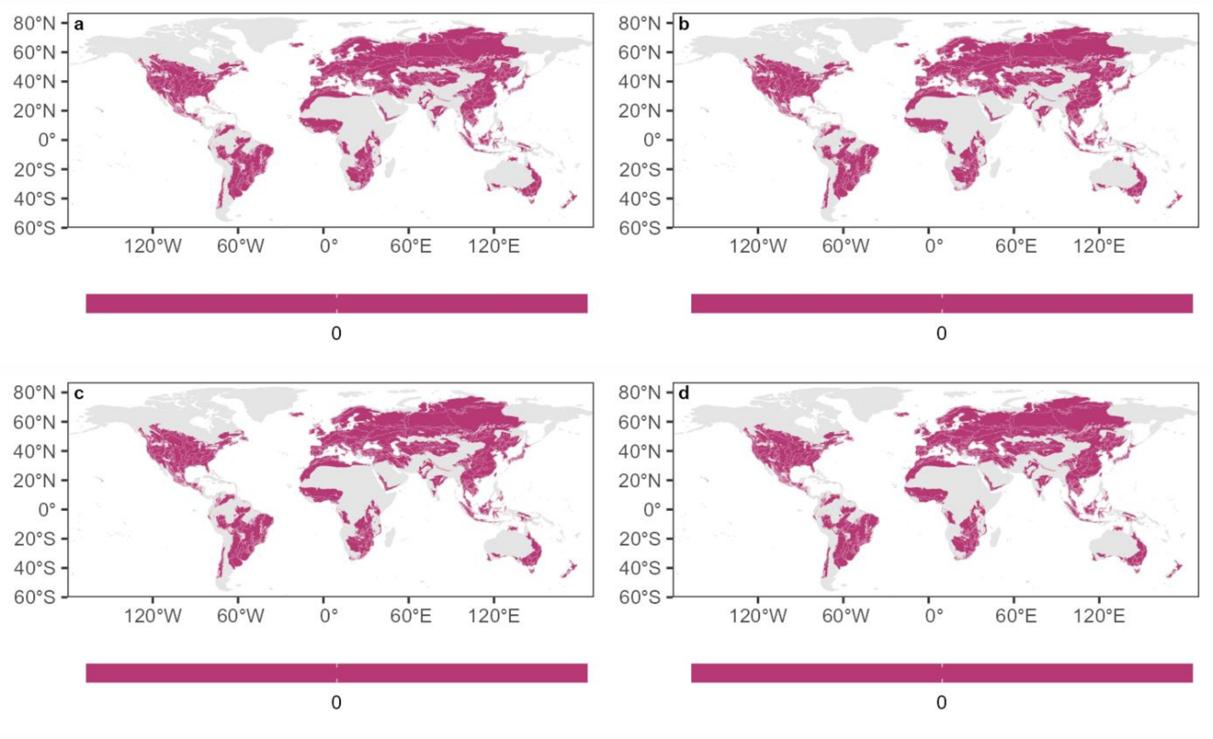


Figure S14. Land occupation characterization factors at the ecoregion level for urban areas with minimal use and potential impacts on plant species richness. The unit is PDF/$m^2$. a) average impacts on regional species richness, b) marginal impacts on regional species richness, c) average impacts on global species richness, d) marginal impacts on global species richness. Grey denotes no data, indicating either the absence of the specific land use class in these regions or missing species data. More characterization factors are available through the use of proxies.

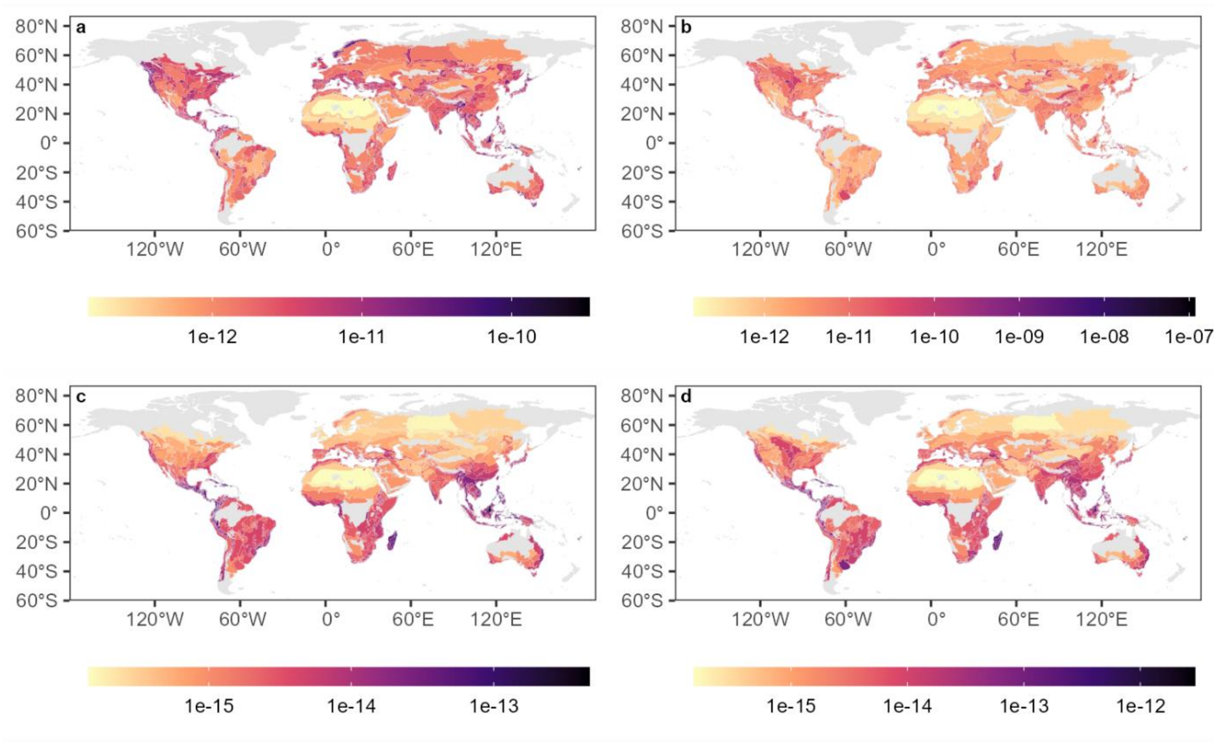


Figure S15. Land occupation characterization factors at the ecoregion level for cropland with intense or light use and potential impacts on vertebrate species richness. The unit is PDF/m$^2$. a) average impacts on regional species richness, b) marginal impacts on regional species richness, c) average impacts on global species richness, d) marginal impacts on global species richness. Grey denotes no data, indicating either the absence of the specific land use class in these regions or missing species data. Note that the impacts of cropland with intense or light use on vertebrate species richness are not differentiated. More characterization factors are available through the use of proxies.

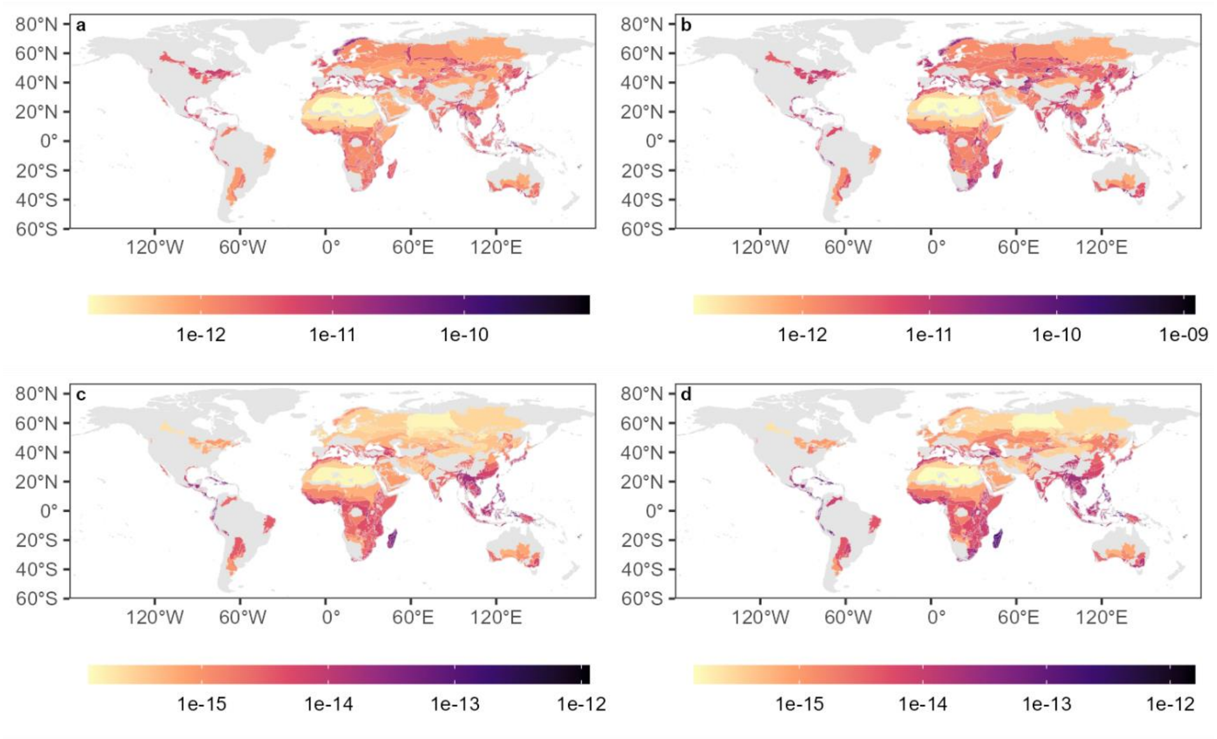


Figure S16. Land occupation characterization factors at the ecoregion level for cropland with minimal use and potential impacts on vertebrate species richness. The unit is PDF/m$^2$. a) average impacts on regional species

richness, b) marginal impacts on regional species richness, c) average impacts on global species richness, d) marginal impacts on global species richness. Grey denotes no data, indicating either the absence of the specific land use class in these regions or missing species data. More characterization factors are available through the use of proxies.

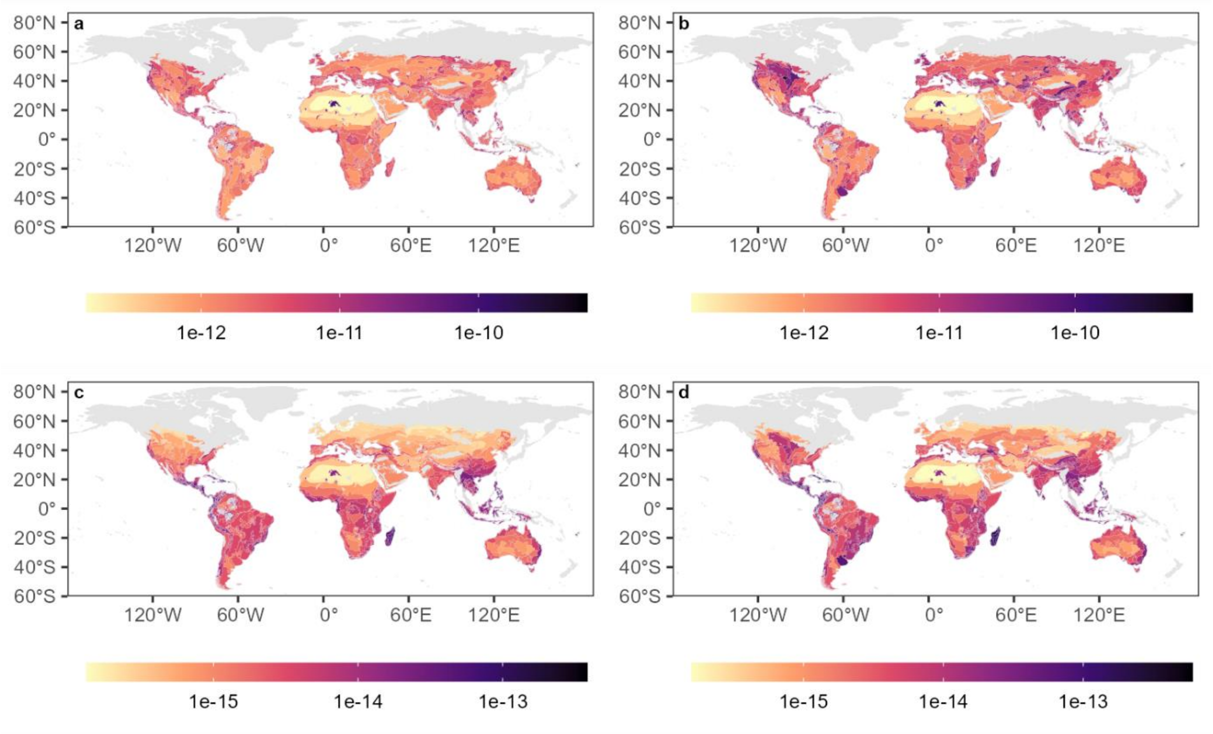


Figure S17. Land occupation characterization factors at the ecoregion level for pasture with intense or light use and potential impacts on vertebrate species richness. The unit is PDF/m$^2$. a) average impacts on regional species richness, b) marginal impacts on regional species richness, c) average impacts on global species richness, d) marginal impacts on global species richness. Grey denotes no data, indicating either the absence of the specific land use class in these regions or missing species data. Note that the impacts of pasture with intense or light use on vertebrate species richness are not differentiated. More characterization factors are available through the use of proxies.

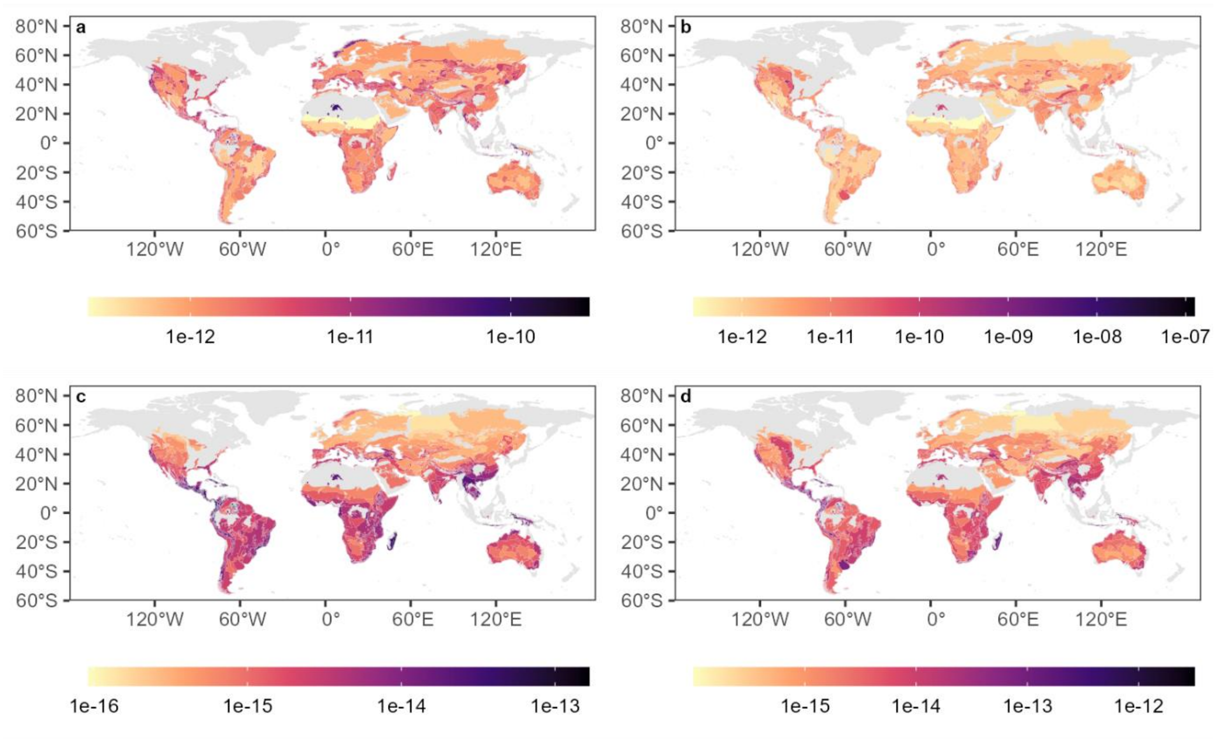


Figure S18. Land occupation characterization factors at the ecoregion level for pasture with minimal use and potential impacts on vertebrate species richness. The unit is PDF/$m^2$. a) average impacts on regional species richness, b) marginal impacts on regional species richness, c) average impacts on global species richness, d) marginal impacts on global species richness. Grey denotes no data, indicating either the absence of the specific land use class in these regions or missing species data. More characterization factors are available through the use of proxies.

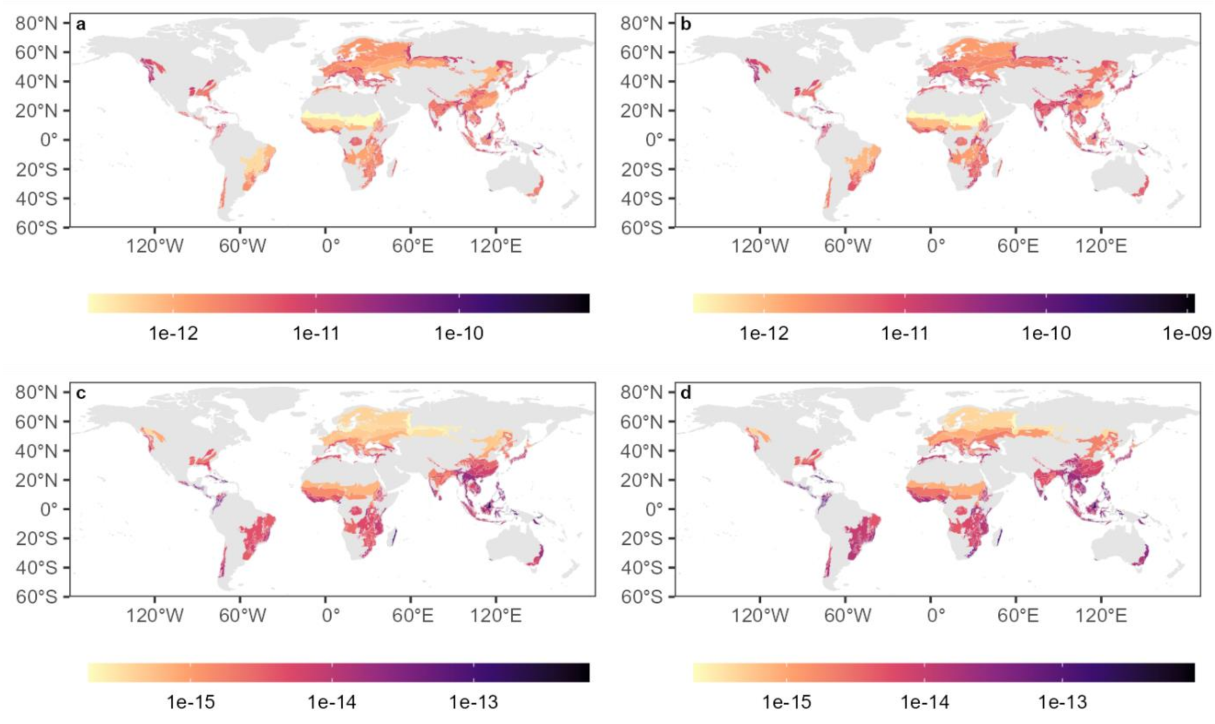


Figure S19. Land occupation characterization factors at the ecoregion level for plantations with intense or light use and potential impacts on vertebrate species richness. The unit is PDF/$m^2$. a) average impacts on regional species richness, b) marginal impacts on regional species richness, c) average impacts on global species richness,

d) marginal impacts on global species richness. Grey denotes no data, indicating either the absence of the specific land use class in these regions or missing species data. Note that the impacts of plantations with intense or light use on vertebrate species richness are not differentiated. More characterization factors are available through the use of proxies.

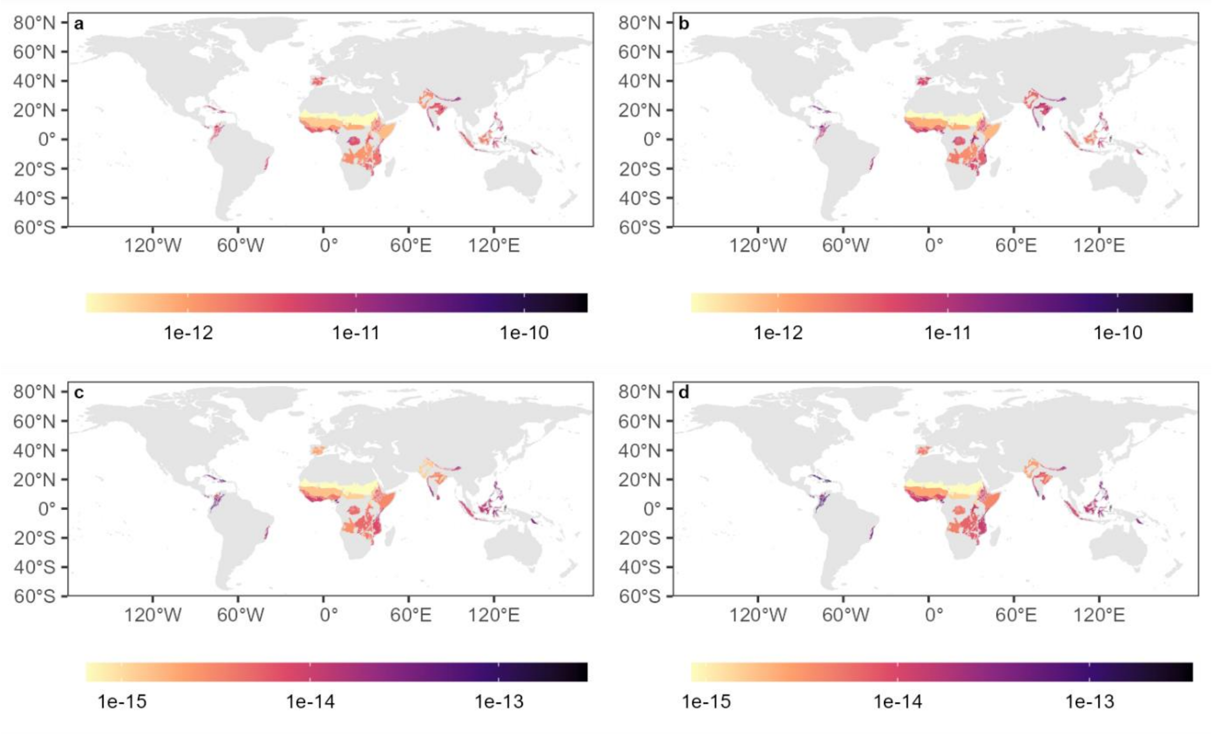


Figure S20. Land occupation characterization factors at the ecoregion level for plantations with minimal use and potential impacts on vertebrate species richness. The unit is PDF/$m^2$. a) average impacts on regional species richness, b) marginal impacts on regional species richness, c) average impacts on global species richness, d) marginal impacts on global species richness. Grey denotes no data, indicating either the absence of the specific land use class in these regions or missing species data. More characterization factors are available through the use of proxies.

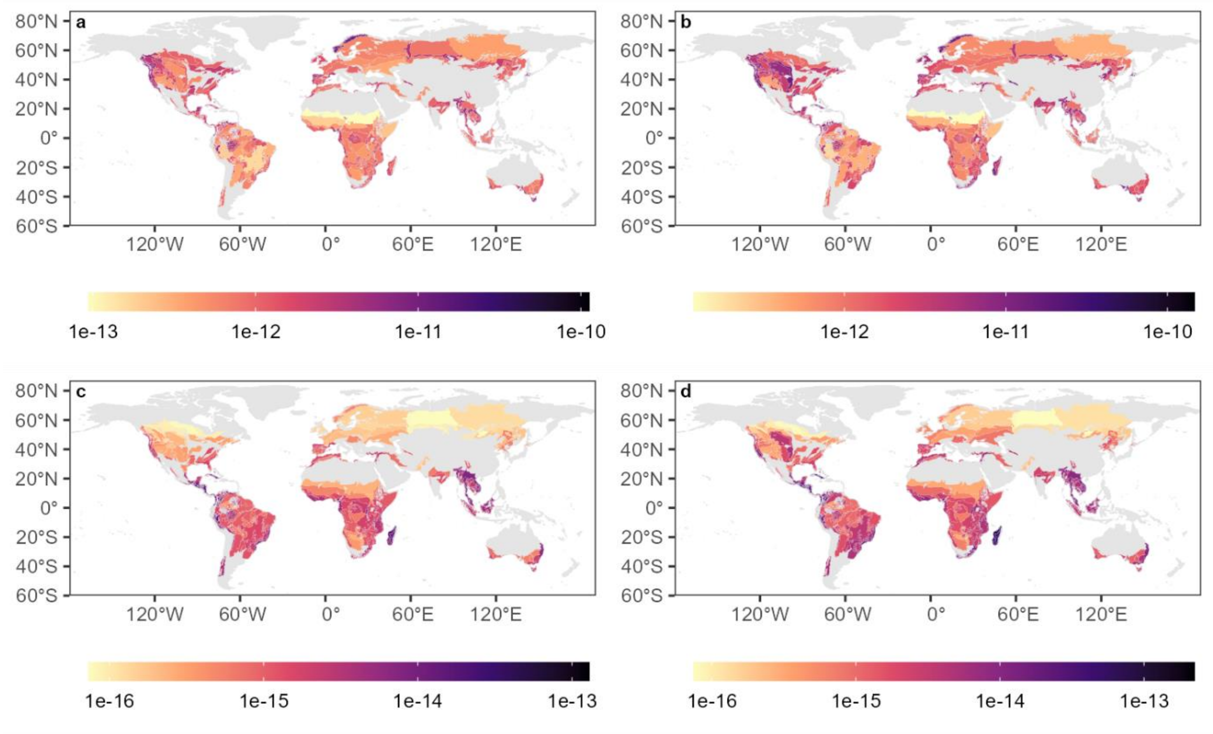


Figure S21. Land occupation characterization factors at the ecoregion level for managed forest with intense use and potential impacts on vertebrate species richness. The unit is PDF/m$^2$. a) average impacts on regional species richness, b) marginal impacts on regional species richness, c) average impacts on global species richness, d) marginal impacts on global species richness. Grey denotes no data, indicating either the absence of the specific land use class in these regions or missing species data. More characterization factors are available through the use of proxies.

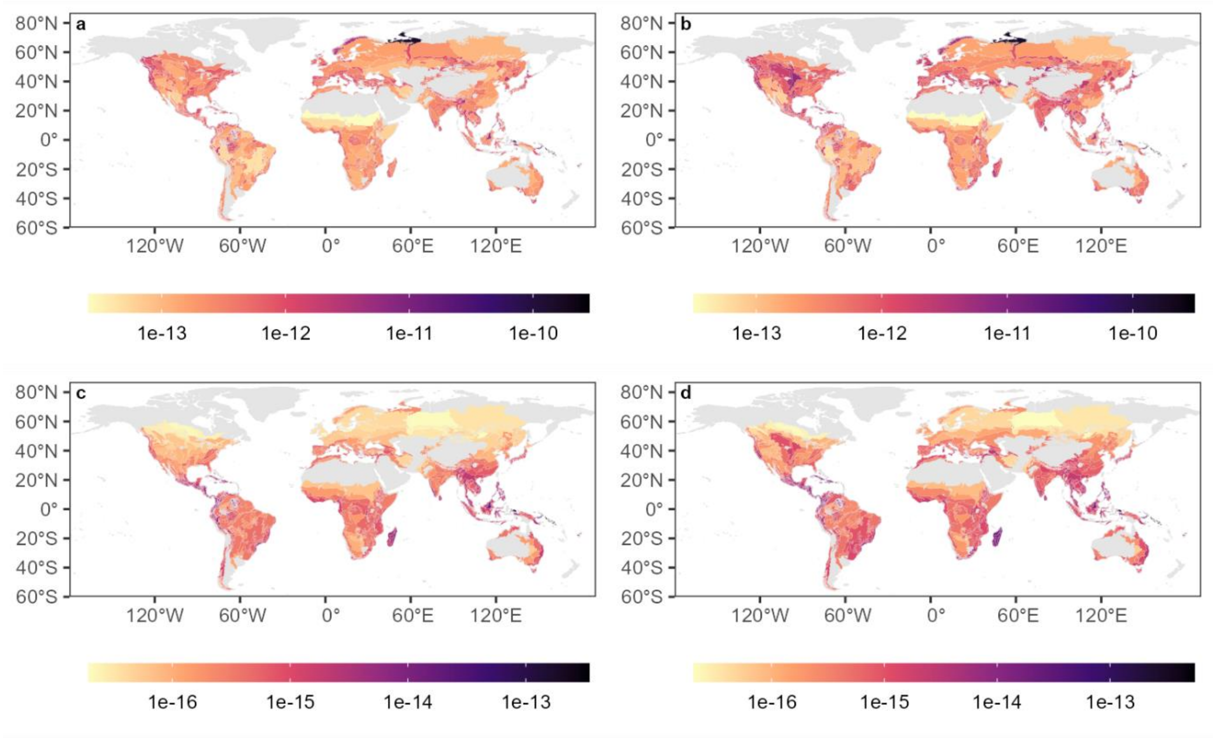


Figure S22. Land occupation characterization factors at the ecoregion level for managed forest with light use and potential impacts on vertebrate species richness. The unit is PDF/m$^2$. a) average impacts on regional species richness, b) marginal impacts on regional species richness, c) average impacts on global species richness, d)

marginal impacts on global species richness. Grey denotes no data, indicating either the absence of the specific land use class in these regions or missing species data. More characterization factors are available through the use of proxies.

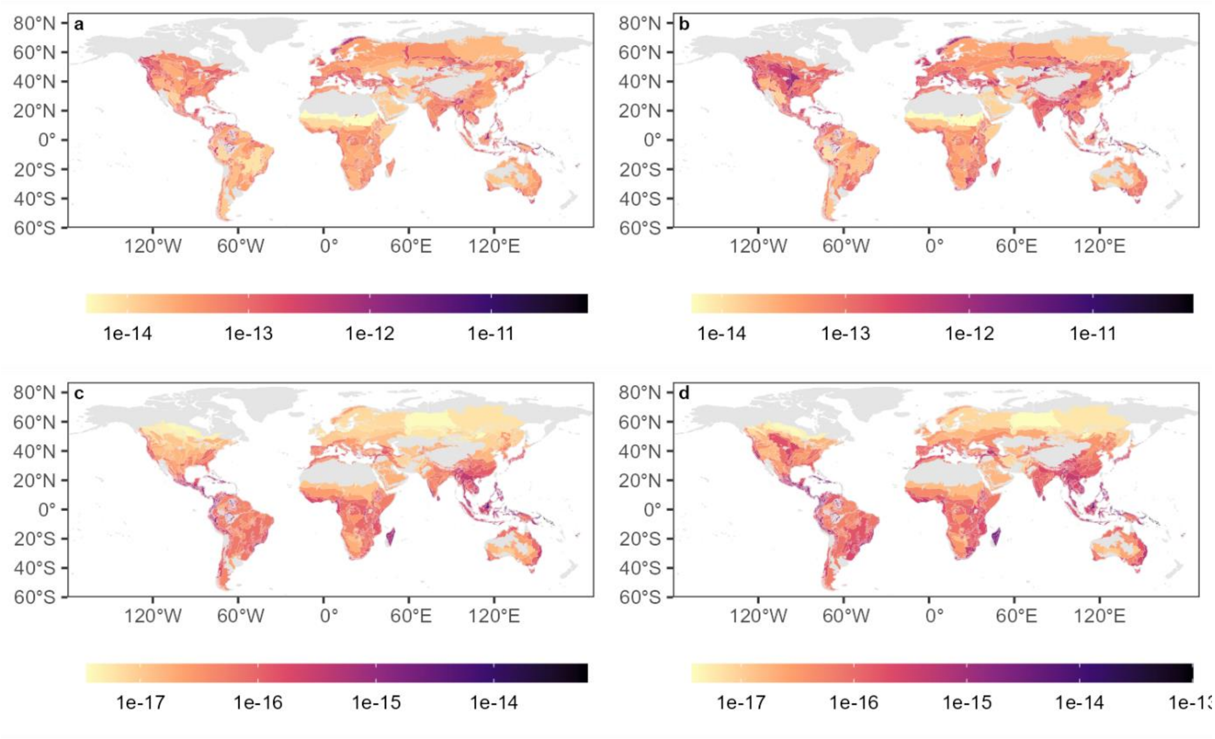


Figure S23. Land occupation characterization factors at the ecoregion level for managed forest with minimal use and potential impacts on vertebrate species richness. The unit is PDF/m$^2$. a) average impacts on regional species richness, b) marginal impacts on regional species richness, c) average impacts on global species richness, d) marginal impacts on global species richness. Grey denotes no data, indicating either the absence of the specific land use class in these regions or missing species data. More characterization factors are available through the use of proxies.

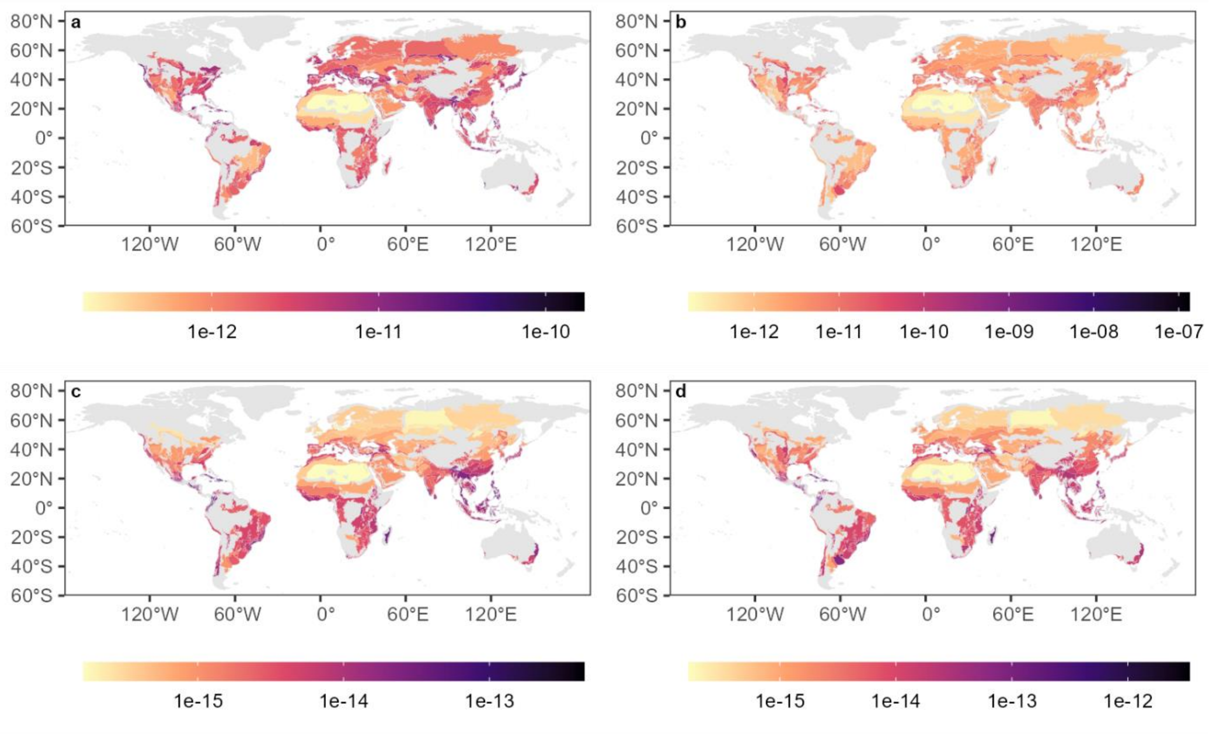


Figure S24. Land occupation characterization factors at the ecoregion level for urban areas with intense use and potential impacts on vertebrate species richness. The unit is PDF/m$^2$. a) average impacts on regional species richness, b) marginal impacts on regional species richness, c) average impacts on global species richness, d) marginal impacts on global species richness. Grey denotes no data, indicating either the absence of the specific land use class in these regions or missing species data. More characterization factors are available through the use of proxies.

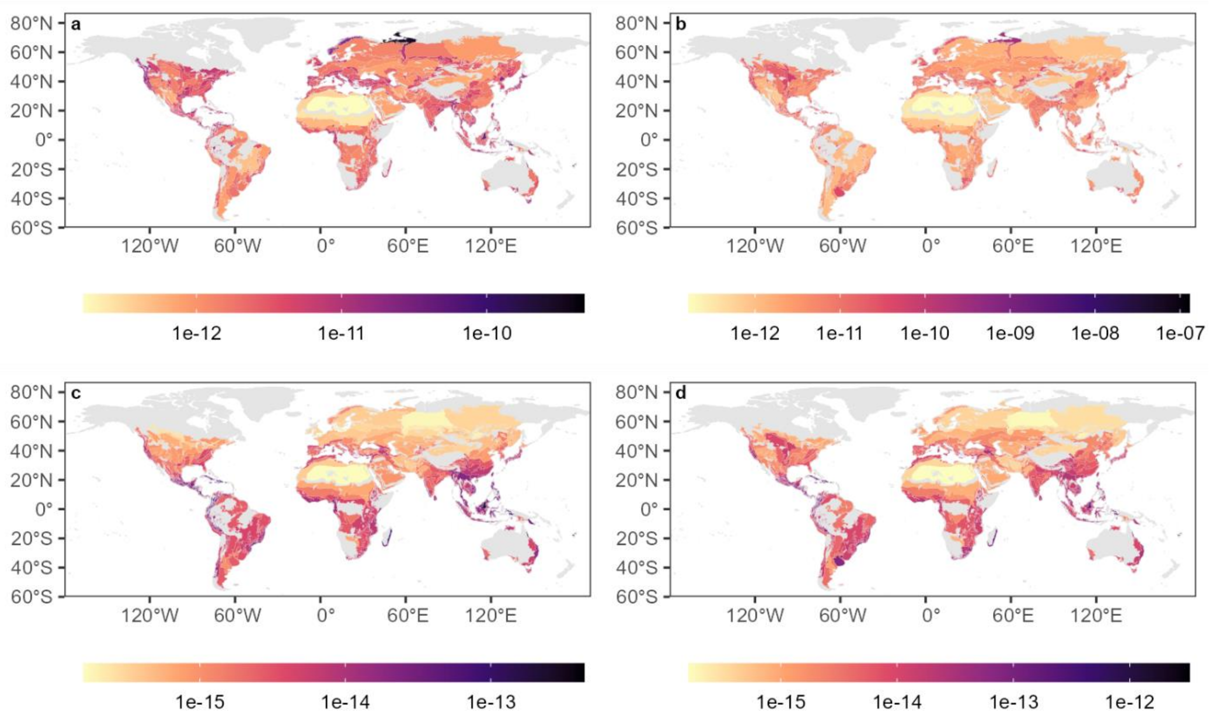


Figure S25. Land occupation characterization factors at the ecoregion level for urban areas with light use and potential impacts on vertebrate species richness. The unit is PDF/m$^2$. a) average impacts on regional species richness, b) marginal impacts on regional species richness, c) average impacts on global species richness, d)

marginal impacts on global species richness. Grey denotes no data, indicating either the absence of the specific land use class in these regions or missing species data. More characterization factors are available through the use of proxies.

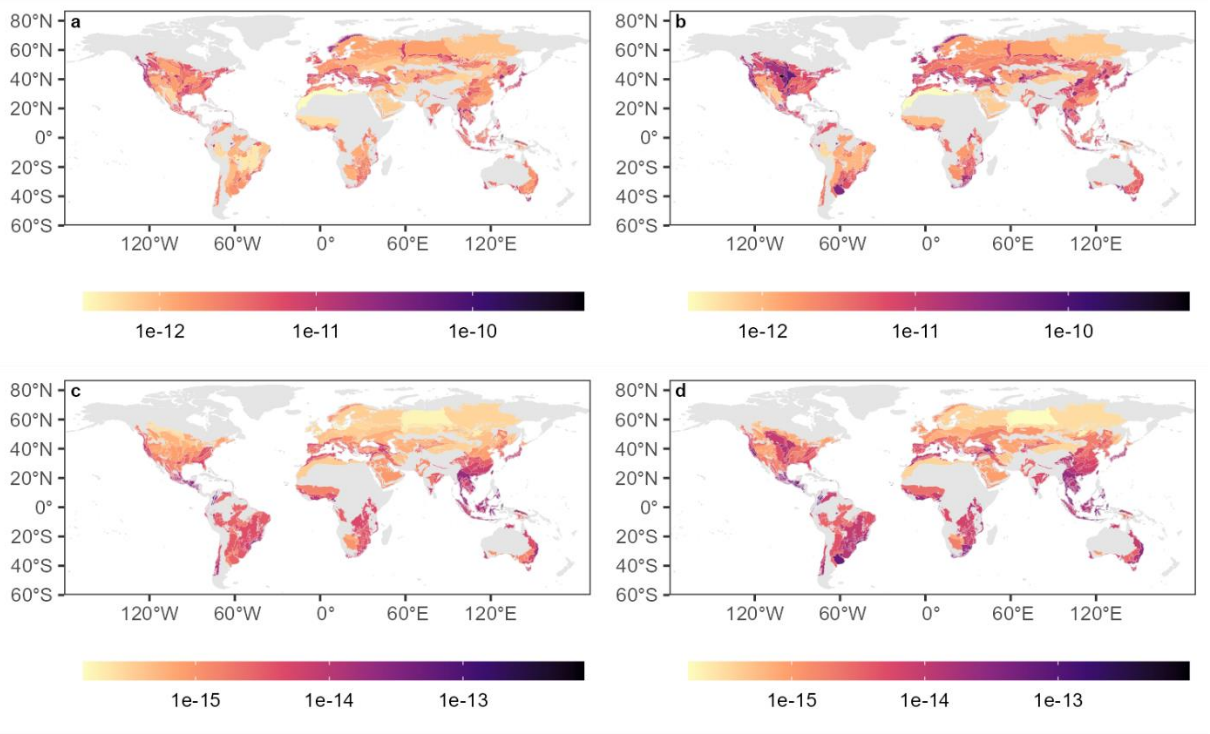


Figure S26. Land occupation characterization factors at the ecoregion level for urban areas with minimal use and potential impacts on vertebrate species richness. The unit is PDF/m$^2$. a) average impacts on regional species richness, b) marginal impacts on regional species richness, c) average impacts on global species richness, d) marginal impacts on global species richness. Grey denotes no data, indicating either the absence of the specific land use class in these regions or missing species data. More characterization factors are available through the use of proxies.

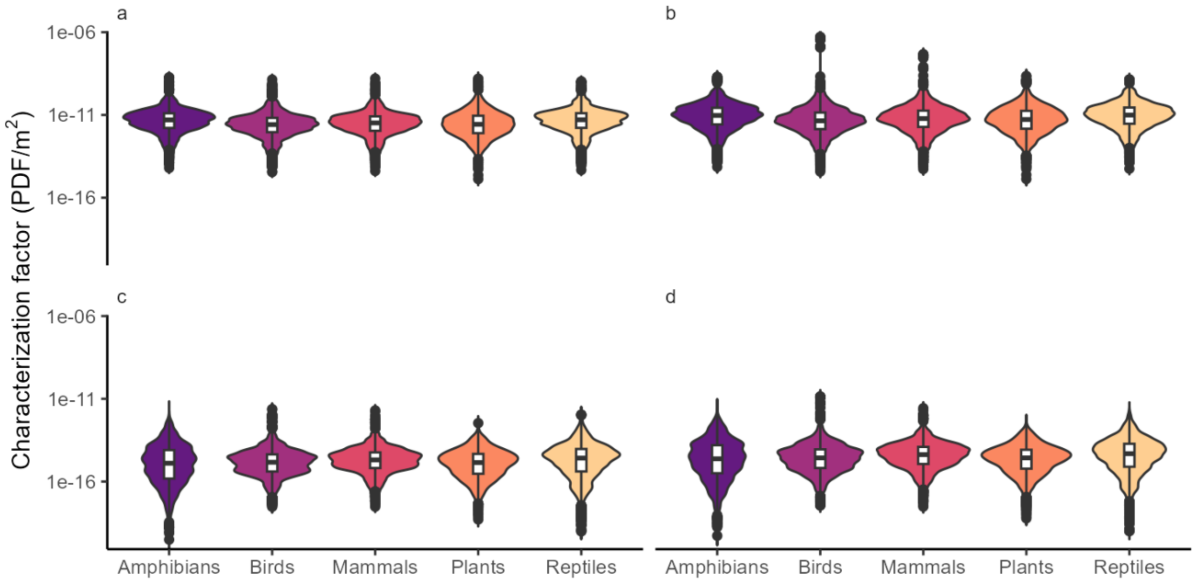


Figure S27. Land occupation characterisation factors for different species groups. a) average impacts on regional species richness, b) marginal impacts on regional species richness, c) average impacts on global species richness, d) marginal impacts on global species richness.

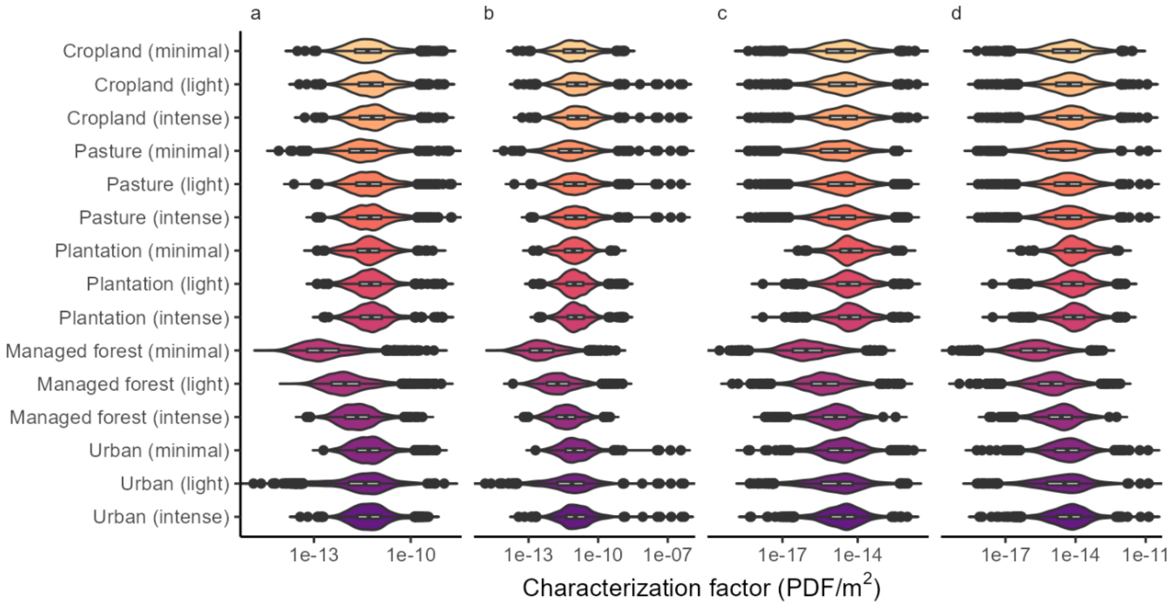


Figure S28. Land occupation characterisation factors for different species groups. a) average impacts on regional species richness, b) marginal impacts on regional species richness, c) average impacts on global species richness, d) marginal impacts on global species richness.

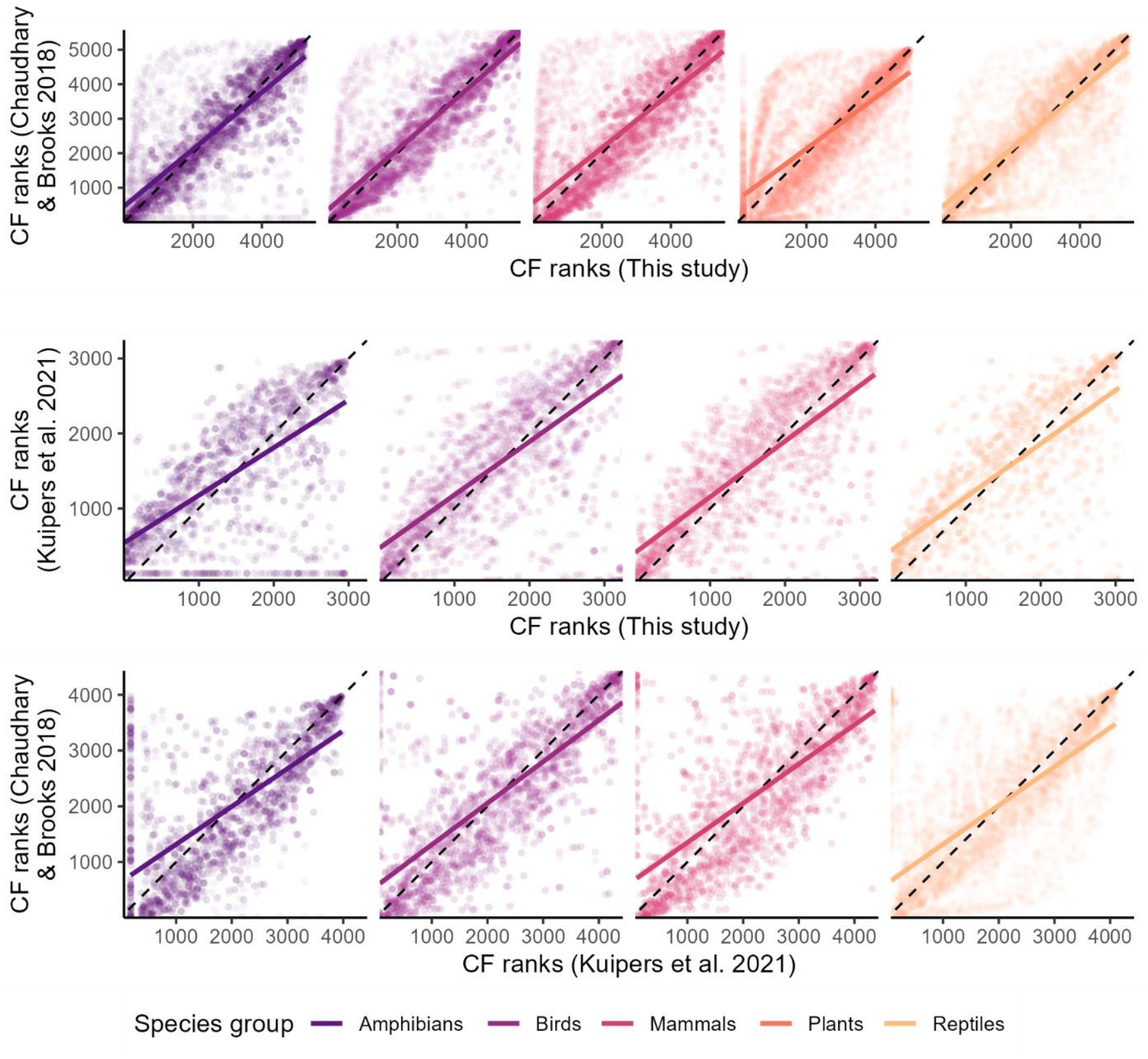


Figure S29. Comparison of characterization factor (CF) ranks from this study and two previous studies across species groups. The black dashed line is the line of equality, while the coloured solid lines are linear trend lines.

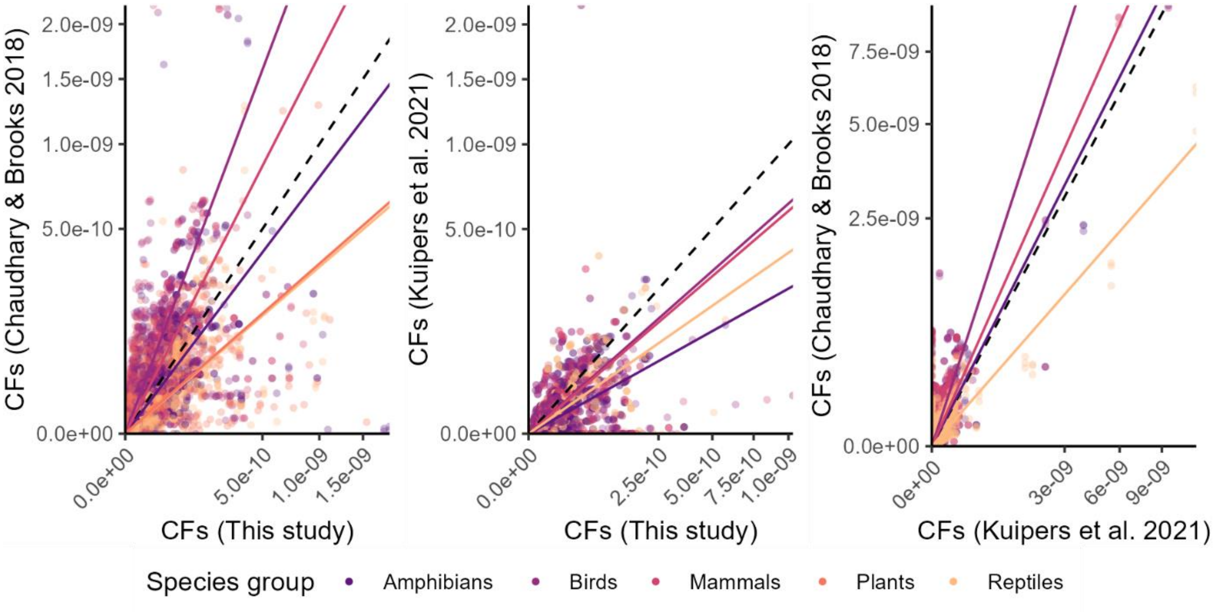


Figure S30. Comparison of characterization factors (CFs) from this study and two previous studies across species groups. The axes are square-root-transformed. The black dashed line is the line of equality, while the coloured solid lines are robust lines using the Theil-Sen estimator.

## References


(1) Saura, S.; Pascual-Hortal, L. A new habitat availability index to integrate connectivity in landscape conservation planning: Comparison with existing indices and application to a case study. *Landscape and Urban Planning* **2007**, *83* (2), 91–103. DOI: 10.1016/j.landurbplan.2007.03.005.

(2) Baan, L. de; Mutel, C. L.; Curran, M.; Hellweg, S.; Koellner, T. Land Use in Life Cycle Assessment: Global Characterization Factors Based on Regional and Global Potential Species Extinction. *Environmental Science & Technology* **2013**, *47* (16), 9281–9290. DOI: 10.1021/es400592q.

(3) Gallego-Zamorano, J.; Huijbregts, M. A.; Schipper, A. M. Changes in plant species richness due to land use and nitrogen deposition across the globe. *Divers Distrib* **2022**, *28* (4), 745–755. DOI: 10.1111/ddi.13476.

(4) Kehoe, L.; Senf, C.; Meyer, C.; Gerstner, K.; Kreft, H.; Kuemmerle, T. Agriculture rivals biomes in predicting global species richness. *Ecography* **2017**, *40* (9), 1118–1128. DOI: 10.1111/ecog.02508.

(5) Palese, R.; Boillat, C.; Loizeau, P.-A. World Flora Online (WFO) - Quality control workflow for an evolving taxonomic backbone. *Biodiversity Information Science and Standards* **2019**.

(6) Kindt, R. WorldFlora: An R package for exact and fuzzy matching of plant names against the World Flora Online taxonomic backbone data. *Appl. Plant. Sci.* **2020**, *8* (9), e11388. DOI: 10.1002/aps3.11388.

(7) Tamme, R.; Götzenberger, L.; Zobel, M.; Bullock, J. M.; Hooftman, D. A. P.; Kaasik, A.; Pärtel, M. Predicting species' maximum dispersal distances from simple plant traits. *Ecology* **2014**, *95* (2), 505–513. DOI: 10.1890/13-1000.1.

(8) Newbold, T.; Hudson, L. N.; Hill, S. L. L.; Contu, S.; Lysenko, I.; Senior, R. A.; Börger, L.; Bennett, D. J.; Choimes, A.; Collen, B.; Day, J.; Palma, A. de; Díaz, S.; Echeverria-

Londoño, S.; Edgar, M. J.; Feldman, A.; Garon, M.; Harrison, M. L. K.; Alhusseini, T.; Ingram, D. J.; Itescu, Y.; Kattge, J.; Kemp, V.; Kirkpatrick, L.; Kleyer, M.; Correia, D. L. P.; Martin, C. D.; Meiri, S.; Novosolov, M.; Pan, Y.; Phillips, H. R. P.; Purves, D. W.; Robinson, A.; Simpson, J.; Tuck, S. L.; Weiher, E.; White, H. J.; Ewers, R. M.; Mace, G. M.; Scharlemann, J. P. W.; Purvis, A. Global effects of land use on local terrestrial biodiversity. *Nature* **2015**, *520* (7545), 45–50. DOI: 10.1038/nature14324.
(9) Newbold, T. *GitHub repository*. https://github.com/timnewbold (accessed July 2022).
(10) Center for International Earth Science Information Network - CIESIN - Columbia University; Information Technology Outreach Services - ITOS - University of Georgia. *Global Roads Open Access Data Set, Version 1 (gROADSv1)*; NASA Socioeconomic Data and Applications Center (SEDAC), 2013. DOI: 10.7927/H4VD6WCT.
(11) Center for International Earth Science Information Network - CIESIN - Columbia University. *Gridded Population of the World, Version 4 (GPWv4): Population Density Adjusted to Match 2015 Revision UN WPP Country Totals, Revision 11*; NASA Socioeconomic Data and Applications Center (SEDAC), 2018. DOI: 10.7927/H4F47M65.
(12) Nelson, A. *Estimated travel time to the nearest city of 50,000 or more people in year 2000*. https://forobs.jrc.ec.europa.eu/products/gam/ (accessed 2022-08-08).
(13) Chaudhary, A.; Burivalova, Z.; Koh, L. P.; Hellweg, S. Impact of Forest Management on Species Richness: Global Meta-Analysis and Economic Trade-Offs. *Scientific Reports* **2016**, *6* (1), 23954. DOI: 10.1038/srep23954.
(14) Winkler, K.; Fuchs, R.; Rounsevell, M.; Herold, M. Global land use changes are four times greater than previously estimated. *Nature Communications* **2021**, *12* (1), 2501. DOI: 10.1038/s41467-021-22702-2.
(15) Mehta, P.; Siebert, S.; Kummu, M.; Deng, Q.; Ali, T.; Marston, L.; Xie, W.; Davis, K. *Majority of 21st century global irrigation expansion has been in water stressed regions*, 2022. DOI: 10.31223/X5C932.
(16) Lu, C.; Tian, H. Global nitrogen and phosphorus fertilizer use for agriculture production in the past half century: shifted hot spots and nutrient imbalance. *Earth Syst. Sci. Data* **2017**, *9* (1), 181–192. DOI: 10.5194/essd-9-181-2017.
(17) Schipper, A. M.; Hilbers, J. P.; Meijer, J. R.; Antão, L. H.; Benítez-López, A.; Jonge, M. M. J. de; Leemans, L. H.; Scheper, E.; Alkemade, R.; Doelman, J. C.; Mylius, S.; Stehfest, E.; van Vuuren, D. P.; van Zeist, W.-J.; Huijbregts, M. A. J. Projecting terrestrial biodiversity intactness with GLOBIO 4. *Glob Change Biol* **2020**, *26* (2), 760–771. DOI: 10.1111/gcb.14848.
(18) Lesiv, M.; Schepaschenko, D.; Buchhorn, M.; See, L.; Dürauer, M.; Georgieva, I.; Jung, M.; Hofhansl, F.; Schulze, K.; Bilous, A.; Blyshchyk, V.; Mukhortova, L.; Brenes, C. L. M.; Krivobokov, L.; Ntie, S.; Tsogt, K.; Pietsch, S. A.; Tikhonova, E.; Kim, M.; Di Fulvio, F.; Su, Y.-F.; Zadorozhniuk, R.; Sirbu, F. S.; Panging, K.; Bilous, S.; Kovalevskii, S. B.; Kraxner, F.; Rabia, A. H.; Vasylyshyn, R.; Ahmed, R.; Diachuk, P.; Kovalevskyi, S. S.; Bungnamei, K.; Bordoloi, K.; Churilov, A.; Vasylyshyn, O.; Sahariah, D.; Tertyshnyi, A. P.; Saikia, A.; Malek, Ž.; Singha, K.; Feshchenko, R.; Prestele, R.; Akhtar, I. u. H.; Sharma, K.; Domashovets, G.; Spawn-Lee, S. A.; Blyshchyk, O.; Slyva, O.; Ilkiv, M.; Melnyk, O.; Sliusarchuk, V.; Karpuk, A.; Terentiev, A.; Bilous, V.; Blyshchyk, K.; Bilous, M.; Bogovyk, N.; Blyshchyk, I.; Bartalev, S.; Yatskov, M.; Smets, B.; Visconti, P.; Mccallum, I.; Obersteiner, M.; Fritz, S. Global forest management data for 2015 at a 100 m resolution. *Scientific Data* **2022**, *9* (1), 199. DOI: 10.1038/s41597-022-01332-3.

(19) Descals, A.; Wich, S.; Meijaard, E.; Gaveau, D. L. A.; Peedell, S.; Szantoi, Z. High-resolution global map of smallholder and industrial closed-canopy oil palm plantations. *Earth Syst. Sci. Data* **2021**, *13* (3), 1211–1231. DOI: 10.5194/essd-13-1211-2021.
(20) Potapov, P.; Hansen, M. C.; Pickens, A.; Hernandez-Serna, A.; Tyukavina, A.; Turubanova, S.; Zalles, V.; Li, X.; Khan, A.; Stolle, F.; Harris, N.; Song, X.-P.; Baggett, A.; Kommareddy, I.; Kommareddy, A. The Global 2000-2020 Land Cover and Land Use Change Dataset Derived From the Landsat Archive: First Results. *Frontiers in Remote Sensing* **2022**, *3*, 856903.
(21) van Asselen, S.; Verburg, P. H. Land cover change or land-use intensification: simulating land system change with a global-scale land change model. *Glob Change Biol* **2013**, *19* (12), 3648–3667. DOI: 10.1111/gcb.12331.
(22) Florczyk, A. J.; Corbane, C.; Ehrlich, D.; Freire, S.; Kemper, T.; Maffenini, L.; Melchiorri, M.; Pesaresi, M.; Politis, P.; Schiavina, M.; Sabo, F.; Zanchetta, L. *GHSL data package 2019, EUR 29788 EN.*
(23) Kuipers, K. J.; May, R.; Verones, F. Considering habitat conversion and fragmentation in characterisation factors for land-use impacts on vertebrate species richness. *Science of The Total Environment* **2021**, *801*, 149737. DOI: 10.1016/j.scitotenv.2021.149737.
(24) Chaudhary, A.; Brooks, T. M. Land Use Intensity-Specific Global Characterization Factors to Assess Product Biodiversity Footprints. *Environmental Science & Technology* **2018**, *52* (9), 5094–5104. DOI: 10.1021/acs.est.7b05570.
