# Supplementary material for: Biodiversity Impact Assessment Considering Land Use Intensities and Fragmentation
Source: Environ Sci Technol. 2023 Nov 16;57(48):19612–23. doi: 10.1021/acs.est.3c04191 (PMC10702493; doi:10.1021/acs.est.3c04191)
Supplement: Supplementary file 2 — es3c04191_si_002.pdf [file es3c04191_si_002.pdf]

Supporting information 2

Biodiversity Impact Assessment Considering Land Use Intensities and Fragmentation

Laura Scherer[1,], Francesca Rosa[2], Zhongxiao Sun[3], Ottar Michelsen[4], Valeria De Laurentiis[5], Alexandra Marques[6], Stephan Pfister[2], Francesca Verones[7], Koen J. J. Kuipers[8]*

[1] Institute of Environmental Sciences (CML), Leiden University, 2333 CC Leiden, The Netherlands

[2] Institute of Environmental Engineering, ETH Zurich, 8093 Zurich, Switzerland

[3] College of Land Science and Technology, China Agricultural University, Beijing 100083, China

[4] Department of Industrial Economics and Technology Management, Norwegian University of Science and Technology (NTNU), 7491 Trondheim, Norway

[5] European Commission-Joint Research Centre, 21027 Ispra, Italy

[6] PBL Netherlands Environmental Assessment Agency, 2500 GH The Hague, The Netherlands

[7] Industrial Ecology Programme, Department for Energy and Process Engineering, Norwegian University of Science and Technology (NTNU), 7491 Trondheim, Norway

[8] Department of Environmental Science, Radboud Institute for Biological and Environmental Sciences (RIBES), Radboud University, 6525AJ Nijmegen, The Netherlands

Summary

11 pages

References of the datasets contributed to TRY that were used in the analysis

Abakumova, M., Zobel K., Lepik, A., Semchenko, M. (2016) Plasticity in plant functional traits is shaped by variability in neighbourhood species composition. New Phytologist 211:455–463 doi:10.1111/nph.13935

Bahn, M., G. Wohlfahrt, E. Haubner, I. Horak, W. Michaeler, K. Rottmar, U. Tappeiner, and A. Cernusca. 1999. Leaf photosynthesis, nitrogen contents and specific leaf area of 30 grassland species in differently managed mountain ecosystems in the Eastern Alps. Pages 247-255 in A. Cernusca, U. Tappeiner, and N. Bayfield, editors. Land-use changes in European mountain ecosystems. ECOMONT- Concept and Results. Blackwell Wissenschaft, Berlin.

Baruch, Z. & Goldstein, G. 1999. Leaf construction cost, nutrient concentration, and net CO2 assimilation of native and invasive species in Hawaii. Oecologia 121: 183-192

Bocanegra-Gonzalez KT, Fermandez-Mendez F, Galvis-Jimenez, JF. (in press) Determinación de la resiliencia en bosques secundarios húmedos tropicales a través de la diversidad funcional de árboles en la región del Bajo Calima, Buenaventura, Colombia.

Brendan Choat, Steven Jansen et al. (2012) Global convergence in the vulnerability of forests to drought. Nature 491, 752–755 doi:10.1038/nature11688

Brendan Choat, Steven Jansen, Tim J. Brodribb, Herve Cochard, Sylvain Delzon, Radika Bhaskar, Sandra J. Bucci, Taylor S. Feild, Sean M. Gleason, Uwe G. Hacke, Anna L. Jacobsen, Frederic Lens, Hafiz Maherali, Jordi Martinez-Vilalta, Stefan Mayr, Maurizio Mencuccini, Patrick J. Mitchell, Andrea Nardini, Jarmila Pittermann, R. Brandon Pratt, John S. Sperry, Mark Westoby, Ian J. Wright & Amy E. Zanne (2012) Global convergence in the vulnerability of forests to drought. Nature 491:752-755 doi:10.1038/nature11688

Bucher, S.F., Auerswald, K., Tautenhahn, S., Geiger, A., Otto, J., Müller, A. and Römermann, C. (2016) Intra- and interspecific variation in stomatal pore area index along altitudinal gradients and its relation to leaf functional traits. Plant Ecology 217, 229-240

Burrascano S, Copiz R, Del Vico E, Fagiani S, Giarrizzo E, Mei M, Mortelliti A, Sabatini FM, Blasi C (2015) Wild boar rooting intensity determines shifts in understorey composition and functional traits. COMMUNITY ECOLOGY 16(2) 244-253 DOI: 10.1556/168.2015.16.2.12

Butterfield, B.J. and J.M. Briggs. 2011. Regeneration niche differentiates functional strategies of desert woody plant species. Oecologia, 165:477-487.

Campbell, C., L. Atkinson, J. Zaragoza-Castells, M. Lundmark, O. Atkin, and V. Hurry. 2007. Acclimation of photosynthesis and respiration is asynchronous in response to changes in temperature regardless of plant functional group. New Phytologist 176:375-389.

Carswell, F. E., Meir, P., Wandelli, E. V., Bonates, L. C. M., Kruijt, B., Barbosa, E. M., Nobre, A. D. & Jarvis, P. G. 2000 Photosynthetic capacity in a central Amazonian rain forest. Tree physiology. 20, 3, p. 179-186 8 p.

Castro-Diez, P., J. P. Puyravaud, J. H. C. Cornelissen, and P. Villar-Salvador. 1998. Stem anatomy and relative growth rate in seedlings of a wide range of woody plant species and types. Oecologia 116:57-66.

Catford, J. A., Morris, W. K., Vesk, P. A., Gippel, C. J. & Downes, B. J. (2014) Species and environmental characteristics point to flow regulation and drought as drivers of riparian plant invasion. Diversity and Distributions, 20, 1084–1096. http://dx.doi.org/10.1111/ddi.12225

Ciccarelli D. (2015) - Mediterranean coastal dune vegetation: Are disturbance and stress the key selective forces that drive the psammophilous succession? Estuarine, Coastal and Shelf Science 165(5):247–253 doi: 10.1016/j.ecss.2015.05.023

Cornelissen, J. H. C. 1996. An experimental comparison of leaf decomposition rates in a wide range of temperate plant species and types. Journal of Ecology 84:573-582.

Cornelissen, J. H. C., B. Cerabolini, P. Castro-Diez, P. Villar-Salvador, G. Montserrat-Marti, J. P. Puyravaud, M. Maestro, M. J. A. Werger, and R. Aerts. 2003. Functional traits of woody plants: correspondence of species rankings between field adults and laboratory-grown seedlings? Journal of Vegetation Science 14:311-322.

Cornelissen, J. H. C., H. M. Quested, D. Gwynn-Jones, R. S. P. Van Logtestijn, M. A. H. De Beus, A. Kondratchuk, T. V. Callaghan, and R. Aerts. 2004. Leaf digestibility and litter decomposability are related in a wide range of subarctic plant species and types. Functional Ecology 18:779-786.

Cornwell, W. K., J. H. C. Cornelissen, K. Amatangelo, E. Dorrepaal, V. T. Eviner, O. Godoy, S. E. Hobbie, B. Hoorens, H. Kurokawa, N. Pérez-Harguindeguy, H. M. Quested, L. S. Santiago, D. A. Wardle, I. J. Wright, R. Aerts, S. D. Allison, P. van Bodegom, V. Brovkin, A. Chatain, T. V. Callaghan, S. Díaz, E. Garnier, D. E. Gurvich, E. Kazakou, J. A. Klein, J. Read, P. B. Reich, N. A. Soudzilovskaia, M. V. Vaieretti, and M. Westoby. 2008. Plant species traits are the predominant control on litter decomposition rates within biomes worldwide. Ecology Letters 11:1065-1071.

Craine JM, Nippert JB, Towne EG, Tucker S, Kembel SW, Skibbe A, McLauchlan KK (2011) Functional consequences of climate-change induced plant species loss in a tallgrass prairie. Oecologia 165: 1109-1117

Craine JM, Ocheltree TW, Nippert JB, Towne EG, Skibbe AM, Kembel SW, Fargione JE (2012) Global diversity of drought tolerance and grassland climate-change resilience. Nature Climate Change

Craine JM, Towne EG, Ocheltree TW, Nippert JB (2012) Community traitscape of foliar nitrogen isotopes reveals N availability patterns in a tallgrass prairie. Plant Soil 356: 395-403

Craine, J. M., A. J. Elmore, M. P. M. Aidar, M. Bustamante, T. E. Dawson, E. A. Hobbie, A. Kahmen, M. C. Mack, K. K. McLauchlan, A. Michelsen, G. B. Nardoto, L. H. Pardo, J. Penuelas, P. B. Reich, E. A. G. Schuur, W. D. Stock, P. H. Templer, R. A. Virginia, J. M. Welker, and I. J. Wright. 2009. Global patterns of foliar nitrogen isotopes and their relationships with climate, mycorrhizal fungi, foliar nutrient concentrations, and nitrogen availability. New Phytologist 183:980-992.

Craven, D., D. Braden, M. S. Ashton, G. P. Berlyn, M. Wishnie, and D. Dent. 2007. Between and within-site comparisons of structural and physiological characteristics and foliar nutrient content of 14 tree species at a wet, fertile site and a dry, infertile site in Panama. Forest Ecology and Management 238:335-346.

De Frutos, Á., Navarro, T., Pueyo, Y., Alados, C.L., 2015. Inferring Resilience to Fragmentation-Induced Changes in Plant Communities in a Semi-Arid Mediterranean Ecosystem. PLoS ONE 10, e0118837. doi:10.1371/journal.pone.0118837

Díaz, S., J. G. Hodgson, K. Thompson, M. Cabido, J. H. C. Cornelissen, A. Jalili, G. Montserrat-Martí, J. P. Grime, F. Zarrinkamar, Y. Asri, S. R. Band, S. Basconcelo, P. Castro-Díez, G. Funes, B. Hamzehee, M. Khoshnevi, N. Pérez-Harguindeguy, M. C. Pérez-Rontomé, F. A. Shirvany, F. Vendramini, S. Yazdani, R. Abbas-Azimi, A. Bogaard, S. Boustani, M. Charles, M. Dehghan, L. de Torres-Espuny, V. Falczuk, J. Guerrero-Campo, A. Hynd, G. Jones, E. Kowsary, F. Kazemi-Saeed, M. Maestro-Martínez, A. Romo-Díez, S. Shaw, B. Siavash, P. Villar-Salvador, and M. R. Zak. 2004. The plant traits that drive ecosystems: Evidence from three continents. Journal of Vegetation Science 15:295-304.

Domingues TF, Meir P, Feldpausch TR, et al. (2010) Co-limitation of photosynthetic capacity by nitrogen and phosphorus in West Africa woodlands. Plant, Cell & Environment (33): 959-980.

Dressler S, M Schmidt, G Zizka (2014) Introducing African Plants—A Photo Guide—An Interactive Photo Data-Base and Rapid Identification Tool for Continental Africa. Taxon 63(5) 1159-1161 DOI: http://dx.doi.org/10.12705/635.26

Dwyer, J. M., R. J. Hobbs, and M. M. Mayfield. 2014. Specific leaf area responses to environmental gradients through space and time. Ecology 95:399-410

eHALOPH - Halophytes Database (version 3.11) Tim Flowers, Joaquim Santos, Moritz Jahns, Brian Warburton and Peter Reed; http://www.sussex.ac.uk/affiliates/halophytes, accessed 2015

eHALOPH - Halophytes Database (version 3.11) Tim Flowers, Joaquim Santos, Moritz Jahns, Brian Warburton and Peter Reed; http://www.sussex.ac.uk/affiliates/halophytes, accessed 2017

Engemann, K., Sandel, B., Boyle, B., Enquist, B. J., Jørgensen, P. M., Kattge, J., McGill, B. J., Morueta-Holme, N., Peet, R. K., Spencer, N. J., Violle, C., Wiser, S. K. and Svenning, J.-C. (2016), A plant growth form dataset for the New World. Ecology, 97: 3243. doi:10.1002/ecy.1569

Fitter, A. H. and H. J. Peat 1994. The Ecological Flora Database. Journal of Ecology 82:415-425.

Fonseca, C. R., J. M. Overton, B. Collins, and M. Westoby. 2000. Shifts in trait-combinations along rainfall and phosphorus gradients. Journal of Ecology 88:964-977.

Forgiarini, C., Souza, A.F., Longhi, S.J., Oliveira, J.M., 2015. In the lack of extreme pioneers: trait relationships and ecological strategies of 66 subtropical tree species. J. Plant Ecol. 8, 359–367. doi:10.1093/jpe/rtu028

Frenette-Dussault, C., Shipley, B., Léger, J.F., Meziane, D. & Hingrat, Y. (2012). Functional structure of an arid steppe plant community reveals similarities with Grime's C-S-R theory. Journal of Vegetation Science 23: 208-222.

Freschet, G. T., J. H. C. Cornelissen, R. S. P. van Logtestijn, and R. Aerts. 2010. Evidence of the 'plant economics spectrum' in a subarctic flora. Journal of Ecology 98:362-373.

Giroldo, Aelton (2016) Pequenas plantas, grandes estrategias: adaptacoes e sobrevivencia no Cerrado. PhD Thesis University of Brasilia. DOI: 10.13140/RG.2.2.34455.16800

Green, W. 2009. USDA PLANTS Compilation, version 1, 09-02-02. (http://bricol.net/downloads/data/PLANTSdatabase/) NRCS: The PLANTS Database (http://plants.usda.gov, 1 Feb 2009). National Plant Data Center: Baton Rouge, LA 70874-74490 USA.

Gutiérrez AG, & Huth A (2012) Successional stages of primary temperate rainforests of Chiloé Island, Chile. Perspectives in plant ecology, systematics and evolution. 14: 243– 256

Han, W. X., J. Y. Fang, D. L. Guo, and Y. Zhang. 2005. Leaf nitrogen and phosphorus stoichiometry across 753 terrestrial plant species in China. New Phytologist 168:377-385.

Hietz, P., Rosner, S., Hietz-Seifert, U. & Wright, S.J. (2017). Wood traits related to size and life history of trees in a Panamanian rainforest. New Phytol., 213, 170-180

HIGUCHI, P.; SILVA, A.C. Araucaria Forest Database. 2013

Iversen CM, McCormack ML, Powell AS, Blackwood CB, Freschet GT, Kattge J, Roumet C, Stover DB, Soudzilovskaia NA, Valverde-Barrantes OJ, van Bodegom PM, Violle C (2017) A global Fine-Root Ecology Database to address belowground challenges in plant ecology. New Phytologist. doi:10.1111/nph.14486.

Joseph, G.S., Seymour, C.L., Cumming, G.S., Cumming, D.H.M., & Mahlangu, Z. 2014. Termite mounds increase functional diversity of woody plants in African savannas. Ecosystems 17: 808–819.

Kattenborn, T., Fassnacht, F. E., & Schmidtlein, S. (2018). Differentiating plant functional types using reflectance: which traits make the difference? Remote Sensing in Ecology and Conservation, 1–15. http://doi.org/10.1002/rse2.86

Kattge, J., W. Knorr, T. Raddatz, and C. Wirth. 2009. Quantifying photosynthetic capacity and its relationship to leaf nitrogen content for global-scale terrestrial biosphere models. Global Change Biology 15:976-991.

Kearsley, E., Verbeeck, H., Hufkens, K., Van de Perre, F., Doetterl, S., Baert, G., Beeckman, H., Boeckx, P., Huygens, D. (2017). Functional community structure of African monodominant Gilbertiodendron dewevrei forest influenced by local environmental filtering. Ecology and Evolution, 7, 295–304

Kirkup, D., P. Malcolm, G. Christian, and A. Paton. 2005. Towards a digital African Flora. Taxon 54:457-466.

Kleyer, M., R. M. Bekker, I. C. Knevel, J. P. Bakker, K. Thompson, M. Sonnenschein, P. Poschlod, J. M. van Groenendael, L. Klimes, J. Klimesova, S. Klotz, G. M. Rusch, Hermy, M.

, D. Adriaens, G. Boedeltje, B. Bossuyt, A. Dannemann, P. Endels, L. Götzenberger, J. G. Hodgson, A.-K. Jackel, I. Kühn, D. Kunzmann, W. A. Ozinga, C. Römermann, M. Stadler, J. Schlegelmilch, H. J. Steendam, O. Tackenberg, B. Wilmann, J. H. C. Cornelissen, O. Eriksson, E. Garnier, and B. Peco. 2008. The LEDA Traitbase: a database of life-history traits of the Northwest European flora. Journal of Ecology 96:1266-1274.

Knauer et al. (2017) Towards physiologically meaningful water-use efficiency estimates from eddy covariance data. Global Change Biology, DOI: 10.1111/gcb.13893

Kühn, I., W. Durka, and S. Klotz. 2004. BiolFlor - a new plant-trait database as a tool for plant invasion ecology. Diversity and Distribution 10 363-365.

Laughlin, D. C., J. J. Leppert, M. M. Moore, and C. H. Sieg. 2010. A multi-trait test of the leaf-height-seed plant strategy scheme with 133 species from a pine forest flora. Functional Ecology 24:493-501.

Laughlin, D.C., P.Z. Fulé, D.W. Huffman, J. Crouse, and E. Laliberte. 2011. Climatic constraints on trait-based forest assembly. Journal of Ecology 99:1489-1499.

Li, R., Zhu, S., Chen, H. Y. H., John, R., Zhou, G., Zhang, D., Zhang, Q. and Ye, Q. (2015), Are functional traits a good predictor of global change impacts on tree species abundance dynamics in a subtropical forest?. Ecol Lett, 18: 1181–1189. doi:10.1111/ele.12497

Li, Y. and Shipley, B. (2018) Community divergence and convergence along experimental gradients of stress and disturbance. Ecology, 99: 775-781. doi:10.1002/ecy.2162

Liebergesell M, Reu B, Stahl U, Freiberg M, Welk E, Kattge J, Cornelissen JHC, Penuelas J, Wirth C (2016) Functional Resilience against Climate-Driven Extinctions - Comparing the Functional Diversity of European and North American Tree Floras. PLoS ONE 11(2): e0148607. doi:10.1371/journal.pone.0148607

Lin Y-S, Medlyn BE, Duursma RA, Prentice IC, Wang H, Baig S, Eamus D, De Dios VR, Mitchell P, Ellsworth DS, De Beeck MO, Wallin G, Uddling J, Tarvainen L, Linderson M-L, Cernusak LA, Nippert JB, Ocheltree TW, Tissue DT, Martin-StPaul NK, Rogers A, Warren JM, De Angelis P, Hikosaka K, Han Q, Onoda Y, Gimeno TE, Barton CVM, Bennie J, Bonal D, Bosc A, Löw M, Macinins-Ng C, Rey A, Rowland L, Setterfield SA, Tausz-Posch S, Zaragoza-Castells J, Broadmeadow MSJ, Drake JE, Freeman M, Ghannoum O, Hutley LB, Kelly JW, Kikuzawa K, Kolari P, Koyama K, Limousin J-M, Meir P, Da Costa ACL, Mikkelsen TN, Salinas N, Sun W, Wingate L, (2015) Optimal stomatal behaviour around the world. Nature Climate Change 5(5): 459-464 DOI: 10.1038/NCLIMATE2550

Lukeš, P., Stenberg, P., Rautiainen, M., Mõttus, M., Vanhatalo, K.M. Optical properties of leaves and needles for boreal tree species in Europe (2013) Remote Sensing Letters, 4 (7), pp. 667-676

Maire V, Ian J. Wright, I. Colin Prentice, Niels H. Batjes, Radika Bhaskar, Peter M. van Bodegom, Will K. Cornwell, David Ellsworth, Ülo Niinemets, Alejandro Ordoñez, Peter B. Reich, Louis S. Santiago (2015). Global soil and climate effects on leaf photosynthetic traits and rates. Global Ecology and Biogeography 24(6): 706-717. Maire V, Wright IJ, Prentice IC, Batjes NH, Bhaskar R, van Bodegom PM, Cornwell WK, Ellsworth D, Niinemets Ü, Ordoñez A, Reich PB, Santiago LS (2015) Data from: Global effects of soil and climate on leaf

photosynthetic traits and rates. Dryad Digital Repository. http://dx.doi.org/10.5061/dryad.j42m7

Marco Moretti and Colin Legg (2009) Combining plant and animal traits to assess community functional responses to disturbance. Ecography 32: 299 309. doi: 10.1111/j.1600-0587.2008.05524.x

Meir, P., Kruijt, B., Broadmeadow, M., Kull, O., Carswell, F. & Nobre, A. 2002 Acclimation of photosynthetic capacity to irradiance in tree canopies in relation to leaf nitrogen concentration and leaf mass per unit area. Plant, Cell and Environment. 25, 3, p. 343-357 15 p.

MENCUCCINI M., 2003. The ecological significance of long distance water transport: short-term regulation and long-term acclimation across plant growth forms. Plant, Cell and Environment, 26:163-182.

Michaletz, S.T., and E.A. Johnson. 2006. A heat transfer model of crown scorch in forest fires. Canadian Journal of Forest Research 36(11): 2839-2851

Milla & Reich 2011 Annals of Botany 107: 455–465, 2011.

Miller JED, Ives AR, Harrison SP, Damschen EI (2018) Early‐ and late‐flowering guilds respond differently to landscape spatial structure. J Ecol. 106:1033–1045. https://doi.org/10.1111/1365-2745.12849

Minden V, M Kleyer (2015): Ecosystem multifunctionality of coastal marshes is determined by key plant traits, Journal of Vegetation Science 26: 651-662

Minden V, Michael Kleyer (2014): Internal and external regulation of plant organ stoichiometry, Plant Biology, 16: 897-907

Minden Vanessa, Michael Kleyer (2011): Testing the effect–response framework: key response and effect traits determining above-ground biomass of salt marshes. Journal of Vegetation Science 22: 387-401

Minden Vanessa, Sandra Andratschke, Janina Spalke, Hanna Timmermann, Michael Kleyer (2012): Plant trait–environment relationships in salt marshes: Deviations from predictions by ecological concepts. Perspectives in Plant Ecology, Evolution and Systematics, 14: 183-192

Moles, A. T., D. S. Falster, M. R. Leishman, and M. Westoby. 2004. Small-seeded species produce more seeds per square metre of canopy per year, but not per individual per lifetime. Journal of Ecology 92:384-396.

Muller, S. C., G. E. Overbeck, J. Pfadenhauer, and V. D. Pillar. 2007. Plant functional types of woody species related to fire disturbance in forest-grassland ecotones. Plant Ecology 189:1-14.

Niinemets, U. 2001. Global-scale climatic controls of leaf dry mass per area, density, and thickness in trees and shrubs. Ecology 82:453-469.

Ogaya, R. and J. Penuelas. 2003. Comparative field study of Quercus ilex and Phillyrea latifolia: photosynthetic response to experimental drought conditions. Environmental and Experimental Botany 50:137-148.

Onoda Y, Wright IJ, Evans JR, Hikosaka K, Kitajima K, Niinemets Ü, Poorter H, Tosesns T, Westoby M. (2017) Physiological and structural tradeoffs underlying the leaf economics spectrum. New Phytologist

Onoda, Y., M. Westoby, P. B. Adler, A. M. F. Choong, F. J. Clissold, J. H. C. Cornelissen, S. Diaz, N. J. Dominy, A. Elgart, L. Enrico, P. V. A. Fine, J. J. Howard, A. Jalili, K. Kitajima, H. Kurokawa, C. McArthur, P. W. Lucas, L. Markesteijn, N. Perez-Harguindeguy, L. Poorter, L. Richards, L. S. Santiago, Jr. E. Sosinski, S. Van Bael, D. I. Warton, I. J. Wright, S. J. Wright, and N. Yamashita. 2011 . Global patterns of leaf mechanical properties. Ecology Letters 14:301-312.

Onstein RE, Richard J. Carter, Yaowu Xing, H. Peter LinderInstitute (2014) Diversification rate shifts in the Cape Floristic Region: The right traits in the right place at the right time. Perspectives in Plant Ecology, Evolution and Systematics 16(6) 331–340 DOI:10.1016/j.ppees.2014.08.002

Ordonez, J. C., P. M. van Bodegom, J. P. M. Witte, R. P. Bartholomeus, J. R. van Hal, and R. Aerts. 2010. Plant Strategies in Relation to Resource Supply in Mesic to Wet Environments: Does Theory Mirror Nature? American Naturalist 175:225-239.

Paula, S., M. Arianoutsou, D. Kazanis, Ç. Tavsanoglu, F. Lloret, C. Buhk, F. Ojeda, B. Luna, J. M. Moreno, A. Rodrigo, J. M. Espelta, S. Palacio, B. Fernández-Santos, P. M. Fernandes, and J. G. Pausas. 2009. Fire-related traits for plant species of the Mediterranean Basin. Ecology 90:1420.

Peco B., de Pablos I., Traba J. , & Levassor C. (2005) The effect of grazing abandonment on species composition and functional traits: the case of dehesa Basic and Applied Ecology, 6(2): 175-183

Penuelas, J., J. Sardans, J. Llusia, S. Owen, J. Carnicer, T. W. Giambelluca, E. L. Rezende, M. Waite, and Ü. Niinemets. 2010. Faster returns on "leaf economics" and different biogeochemical niche in invasive compared with native plant species. Global Change Biology 16:2171-2185.

Petter G, Wagner K, Zotz G, Cabral JS, Wanek W, Sanchez Delgado EJ, Kreft H. 2016. Distribution of functional leaf traits of vascular epiphytes: vertical trends, intra- and interspecific trait variability, and phylogenetic signals. Functional Ecology, 30: 188–198.

Pisek, J., Sonnentag, O., Richardson, A.D., Mõttus, M. (2013). Is the spherical leaf inclination angle distribution a valid assumption for temperate and boreal broadleaf tree species? Agricultural and Forest Meteorology, 169, 186 - 194

Prentice, I.C., Meng, T., Wang, H., Harrison, S.P., Ni, J., Wang, G., 2011. Evidence for a universal scaling relationship of leaf CO2 drawdown along a moisture gradient. New Phytologist 190: 169–180

Quested, H. M., J. H. C. Cornelissen, M. C. Press, T. V. Callaghan, R. Aerts, F. Trosien, P. Riemann, D. Gwynn-Jones, A. Kondratchuk, and S. E. Jonasson. 2003. Decomposition of sub-arctic plants with differing nitrogen economies: A functional role for hemiparasites. Ecology 84:3209-3221.

Raabe, K., Pisek, J., Sonnentag, O., Annuk, K. (2015). Variations of leaf inclination angle distribution with height over the growing season and light exposure for eight broadleaf tree species. Agricultural and Forest Meteorology, 214-215, 2-11

Reich, P. B., J. Oleksyn, and I. J. Wright. 2009. Leaf phosphorus influences the photosynthesis-nitrogen relation: a cross-biome analysis of 314 species. Oecologia 160:207-212.

Reich, P. B., M. G. Tjoelker, K. S. Pregitzer, I. J. Wright, J. Oleksyn, and J. L. Machado. 2008. Scaling of respiration to nitrogen in leaves, stems and roots of higher land plants. Ecology Letters 11:793-801.

Rolo V., López-Díaz M. L. and Moreno G. (2012) Shrubs affect soil nutrients availability with contrasting consequences for pasture understory and tree overstory production and nutrient status in Mediterranean grazed open woodlands. Nutrient Cycling in Agroecosystems, 1–14

Royal Botanical Gardens KEW. 2008. Seed Information Database (SID). Version 7.1. Available from: http://data.kew.org/sid/ (May 2008).

Scherer-Lorenzen, M., Schulze, E.-D., Don, A., Schumacher, J. & Weller, E. (2007) Exploring the functional significance of forest diversity: A new long-term experiment with temperate tree species (BIOTREE). Perspectives in Plant Ecology, Evolution and Systematics, 9, 53-70.

Schweingruber, F.H., Landolt, W.: The Xylem Database. Swiss Federal Research Institute WSL Updated (2005)

Shiodera, S., J. S. Rahajoe, and T. Kohyama. 2008. Variation in longevity and traits of leaves among co-occurring understorey plants in a tropical montane forest. Journal of Tropical Ecology 24:121-133.

Shipley B., 2002. Trade-offs between net assimilation rate and specific leaf area in determining relative growth rate: relationship with daily irradiance, Functional Ecology(16) 682-689

Shipley, B. and T. T. Vu. 2002. Dry matter content as a measure of dry matter concentration in plants and their parts. New Phytologist 153:359-364.

Siefert, A., Fridley, J.D., and Ritchie, M.E. 2014. Community functional responses to soil and climate at multiple spatial scales: when does intraspecific variation matter? PLOS ONE 9: e111189

Slot, M. and Winter, K. (2017), In situ temperature response of photosynthesis of 42 tree and liana species in the canopy of two Panamanian lowland tropical forests with contrasting rainfall regimes. New Phytol, 214: 1103-1117. doi:10.1111/nph.14469

Slot, M., Rey-Sanchez, C., Winter, K. and Kitajima, K. (2014) Trait-based scaling of temperature-dependent foliar respiration in a species-rich tropical forest canopy. Functional Ecology 28: 1074–1086 doi:10.1111/1365-2435.12263

Smith, N. G. and Dukes, J. S. (2017), LCE: leaf carbon exchange data set for tropical, temperate, and boreal species of North and Central America. Ecology, 98: 2978. doi:10.1002/ecy.1992

Sophie Gachet, Errol Véla, Thierry Tatoni, 2005, BASECO: a floristic and ecological database of Mediterranean French flora. Biodiversity and Conservation 14(4):1023-1034

SOUZA K, HIGUCHI P, SILVA AC, SCHIMALSKI MB, LOEBENS R, BUZZI JUNIOR F, SOUZA CC, RODRIGUES JUNIOR LC, WALTER FF, MISSIO FF, DALLA ROSA A. (2017) Partição de nicho por grupos funcionais de espécies arbóreas em uma floresta subtropical. Rodriguésia [online] vol.68, n.4, pp.1165-1175. ISSN 0370-6583. http://dx.doi.org/10.1590/2175-7860201768401

Spasojevic, M. J., Turner, B. L., and Myers, J. A. (2016) When does intraspecific trait variation contribute to functional beta?diversity? J Ecol, 104: 487-496. doi:10.1111/1365-2745.12518

Swenson, N.G., P. Anglada-Cordero, and J.A. Barone. 2011. Deterministic tropical tree community turnover: evidence from patterns of functional beta diversity along an elevational gradient. Proceedings of the Royal Society B 278:877-884.

Takkis, K. 2014. Changes in plant species richness and population performance in response to habitat loss and fragmentation. DISSERTATIONES BIOLOGICAE UNIVERSITATIS TARTUENSIS 255, 2014-04-07. Available from: http://hdl.handle.net/10062/39546

Takkis, K., Saar, L., Pärtel, M., Helm, A. Effect of environment and landscape on the traits of six plant species in fragmented grasslands. (in preparation)

The Tree of Sex Consortium, Ashman T-L, Bachtrog D, Blackmon H, Goldberg EE, Hahn MW, Kirkpatrick M, Kitano J, Mank JE, Mayrose I, Ming R, Otto SP, Peichel CL, Pennell MW, Perrin N, Ross L, Valenzuela N, Vamosi JC (2014) Tree of sex: a database of sexual systems. Scientific Data 1:140015. http://dx.doi.org/10.1038/sdata.2014.15

Thomas, S.C. and A.R. Martin, 2012. Wood carbon content database. Dryad Repository: http://dx.doi.org/10.5061/dryad.69sg2

unpub.

van de Weg MJ, Meir P,Grace J, Atkin O (2009) Altitudinal variation in leaf mass per unit area, leaf tissue density and foliar nitrogen and phosphorus content along the Amazon-Andes gradient in Peru, Plant Ecology& Diversity, 2: 3, 243-254

van de Weg MJ, Patrick Meir John Grace, Guilmair Damian Ramos (2011) Photosynthetic parameters, dark respiration and leaf traits in the canopy of a Peruvian tropical montane cloud forest Oecologia DOI 10.1007/s00442-011-2068-z

van der Plas, F. & Olff, H. (2014) Mesoherbivores affect grasshopper communities in a megaherbivore-dominated South African savannah. Oecologia 175: 639. doi:10.1007/s00442-014-2920-z

Vergutz, L., S. Manzoni, A. Porporato, R.F. Novais, and R.B. Jackson. 2012. A Global Database of Carbon and Nutrient Concentrations of Green and Senesced Leaves. Data set. Available on-line [http://daac.ornl.gov] from Oak Ridge National Laboratory Distributed

Active Archive Center, Oak Ridge, Tennessee, U.S.A. http://dx.doi.org/10.3334/ORNLDAAC/1106

Von Holle, B. and D. Simberloff. 2004. Testing Fox's assembly rule: Does plant invasion depend on recipient community structure? Oikos 105:551-563.

Walker, A.P. 2014. A Global Data Set of Leaf Photosynthetic Rates, Leaf N and P, and Specific Leaf Area. Data set. Available on-line [http://daac.ornl.gov] from Oak Ridge National Laboratory Distributed Active Archive Center, Oak Ridge, Tennessee, USA. http://dx.doi.org/10.3334/ORNLDAAC/1224

Wang, Han; Harrison, Sandy P; Prentice, Iain Colin; Yang, Yanzheng; Bai, Fan; Furstenau Togashi, Henrique; Wang, Meng; Zhou, Shuangxi; Ni, Jian (2017): The China Plant Trait Database. PANGAEA, https://doi.org/10.1594/PANGAEA.871819

Wenxuan Han, Yahan Chen, Fang-Jie Zhao, Luying Tang, Rongfeng Jiang and Fusuo Zhang, 2012, Floral, climatic and soil pH controls on leaf ash content in China's terrestrial plants. Global Ecology and Biogeography, DOI: 10.1111/j.1466-8238.2011.00677.x

White, M. A., P. E. Thornton, S. W. Running, and R. R. Nemani. 2000. Parameterization and sensitivity analysis of the BIOME-BGC terrestrial ecosystem model: Net primary production controls. Earth Interactions 4:1-85.

Wirth, C. and J. W. Lichstein. 2009. The Imprint of Species Turnover on Old-Growth Forest Carbon Balances - Insights From a Trait-Based Model of Forest Dynamics. Pages 81-113 in C. Wirth, G. Gleixner, and M. Heimann, editors. Old-Growth Forests: Function, Fate and Value. Springer, New York, Berlin, Heidelberg.

Wright, I. J., D. D. Ackerly, F. Bongers, K. E. Harms, G. Ibarra-Manriquez, M. Martinez-Ramos, S. J. Mazer, H. C. Muller-Landau, H. Paz, N. C. A. Pitman, L. Poorter, M. R. Silman, C. F. Vriesendorp, C. O. Webb, M. Westoby, and S. J. Wright. 2007. Relationships among ecologically important dimensions of plant trait variation in seven Neotropical forests. Annals of Botany 99:1003-1015.

Wright, I. J., N. Dong, V. Maire, I. C. Prentice, M. Westoby, S. Díaz, R. V. Gallagher, B. F. Jacobs, R. Kooyman, E. A. Law, M. R. Leishman, Ü. Niinemets, P. B. Reich, L. Sack, R. Villar, H. Wang and P. Wilf (2017). Global climatic drivers of leaf size. Science 357(6354): 917-921. DOI:10.1126/science.aal4760

Wright, I. J., P. B. Reich, M. Westoby, D. D. Ackerly, Z. Baruch, F. Bongers, J. Cavender-Bares, T. Chapin, J. H. C. Cornelissen, M. Diemer, J. Flexas, E. Garnier, P. K. Groom, J. Gulias, K. Hikosaka, B. B. Lamont, T. Lee, W. Lee, C. Lusk, J. J. Midgley, M. L. Navas, U. Niinemets, J. Oleksyn, N. Osada, H. Poorter, P. Poot, L. Prior, V. I. Pyankov, C. Roumet, S. C. Thomas, M. G. Tjoelker, E. J. Veneklaas, and R. Villar. 2004. The worldwide leaf economics spectrum. Nature 428:821-827.

Wright, S. J., K. Kitajima, N. J. B. Kraft, P. B. Reich, I. J. Wright, D. E. Bunker, R. Condit, J. W. Dalling, S. J. Davies, S. Díaz, B. M. J. Engelbrecht, K. E. Harms, S. P. Hubbell, C. O. Marks, M. C. Ruiz-Jaen, C. M. Salvador, and A. E. Zanne. 2011 . Functional traits and the growth-mortality tradeoff in tropical trees. Ecology 91:3664-3674.
